# BRD4 Signaling Maintains the Differentiated State of β Cells

**DOI:** 10.1002/advs.202505659

**Published:** 2025-06-20

**Authors:** Fuqiang Liu, Guang Liu, Jia Song, Yujing Sun, Mengmeng Yang, Hualin Liu, Hongkai Zhao, Jiamu Chen, Qincheng Qiao, Siyue Li, Chenglong Yu, Jingru Qu, Ying Zou, Tixiao Wang, Jidong Liu, Lei Zhao, Huihui Tian, Tao Huang, Manna Zhang, Li Chen, Ruxing Zhao, Yuanyuan Du, Xinguo Hou

**Affiliations:** ^1^ Department of Endocrinology and Metabolism Qilu Hospital of Shandong University Jinan Shandong 250012 China; ^2^ Shandong Provincial Key Laboratory of Spatiotemporal Regulation and Precision Intervention in Endocrine and Metabolic Diseases Shandong Provincial Engineering Research Center for Advanced Technologies in Prevention and Treatment of Chromic Metabolic Diseases Institute of Endocrine and Metabolic Diseases of Shandong University Jinan Shandong 250012 China; ^3^ Department of Vascular Surgery Shanghai Ninth People's Hospital Shanghai Jiao Tong University School of Medicine Shanghai 200011 China; ^4^ Hangzhou Institute of Medicine (HIM) Chinese Academy of Sciences Hangzhou Zhejiang 310006 China; ^5^ Institute of Women Children and Reproductive Health Shandong University Jinan Shandong 250012 China; ^6^ Department of General Practice Shanghai Tenth People's Hospital Tongji University School of Medicine Shanghai China 200072; ^7^ The First Clinical Medical College Cheeloo College of Medicine Shandong University Jinan 250012 China; ^8^ Research Center for Basic Medical Sciences Qilu Hospital of Shandong University Jinan China 250012; ^9^ Jinan AXZE Medical Test Laboratory Jinan Shandong Province 250101 China

**Keywords:** ATF5, BRD4, β cell, diabetes, differentiation

## Abstract

In diabetes, pancreatic β cells degenerate from their mature differentiated state to a dedifferentiated state. BRD4 plays a pivotal role during embryogenesis and cancer development, but its function in modulating β‐cell differentiation remains unknown. In this study, multiple models including calorie restriction db/db mouse, long‐term and acute conditional knockout mouse, and human islet organoids are adopted to assess BRD4 function in β cells. Two hundred twenty‐two young patients with diabetes are also recruited for whole exome sequencing (WES) to screen for *BRD4* mutations. This study shows that *BRD4* expression is significantly reduced in human diabetic β cells while significantly increased after calorie restriction in the diabetic mouse. β cell differentiation is impaired after long‐term and acute *Brd4* knockout. *BRD4* knockdown in human islet organoids results in the loss of differentiation and reduction of insulin synthesis. It is found that p.R749C can significantly affect BRD4 signaling and might play roles in diabetes development in patients. This study also shows that ATF5 is a direct target of the BRD4 pathway in β cells. Targeting BRD4‐mediated regulatory networks may hold promise for developing novel therapeutic strategies to maintain the differentiated state of β cells.

## Graphical abstract

The comprehensive exploration revealed the critical role of BRD4 in β cells. BRD4 plays a fundamental role in maintaining β cell differentiation because both long‐term and acute BRD4 deficiency result in a reduction in insulin secretion and downregulation of differentiation markers. This study also shows that ATF5 is a functional target in the BRD4 pathway and might be a potential target for BRD4 signaling intervention.

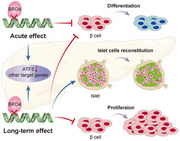

## 1 Introduction

The pancreas, particularly its insulin‐secreting β cells, is central to glucose homeostasis and diabetes pathophysiology.^[^ ^1^ ^]^ Pancreatic β cells respond to fluctuations in blood glucose levels by secreting insulin, a hormone essential for glucose uptake and utilization by peripheral tissues. Mechanistically, in diabetes, β cells degenerate from their mature differentiated state to a dedifferentiated state through the downregulation of β‐cell‐enriched genes, such as *Glut2* (*Slc2a2*), *Pdx1*, and *Mafa*,^[^ ^2^ ^]^ but the underlying mechanism remains less clear. BRD4 is a member of the bromodomain and extraterminal (BET) protein family (BRD2, BRD3, BRD4, and BRDT) and is characterized by two tandem bromodomains (BD1 and BD2). BDs bind acetylated lysine residues on target proteins, including histones.^[^ ^3^ ^]^ BDs function primarily as readers of acetylated lysine residues on histone tails; however, recent evidence indicates that BRD4 has acetylation‐independent transcriptional functions as well as a non‐transcriptional function in controlling DNA damage checkpoint activation and repair.^[^ ^3^, ^4^, ^5^, ^6^ ^]^ Through its interaction with transcription factors, coactivators, and chromatin‐modifying enzymes, BRD4 plays a crucial role in embryogenesis and cancer development. The BET inhibitors JQ1 and I‐BET, which compete with acetylation sites for histone binding to the BDs of the BET protein, were developed and exhibited anti‐proliferative effects in various types of cancer.^[^ ^7^, ^8^ ^]^ The discovery of BET inhibitors has made BRD4 a potential target for the treatment of a variety of diseases, including cancer and inflammation.

However, the roles of BRD4 in pancreatic β cells remain elusive. JQ1 reportedly increases insulin secretion and β‐cell insulin content in vitro.^[^ ^9^ ^]^ I‐BET151 can promote the regeneration of β cells, inducing the proliferation and expression of genes encoding transcription factors which are important for β‐cell differentiation/function in NOD mice.^[^ ^10^ ^]^ It was also reported that chronic treatment with JQ1 or I‐BET151 activates the β‐cell lineage but inhibits insulin gene expression,^[^ ^11^ ^]^ suggesting that BRD4 cannot finalize β‐cell maturation. Recently, one laboratory reported that after JQ1 treatment, insulin secretion was reduced during both in vivo glucose tolerance testing and ex vivo incubation of isolated islets.^[^ ^12^ ^]^ These contradictory findings may be attributed to several factors. First, a major concern in the bromodomain inhibitor field is that these inhibitors are not strongly selective among the BET isoforms BRD2/3/4, and the functions of all three BRDs could be affected simultaneously during JQ1/I‐BET treatment. Second, for in vitro studies in primary islets, inhibitors not only target β cells but also α/δ cells, which might affect paracrine regulation in different cell types. Third, global treatment with JQ1/I‐BET might result in secondary effects on β cell function in vivo. Fourth, the treatment duration and dose of JQ1/I‐BET might impact the final outcome of the experiments. To answer these questions, in this study, two conditional β‐cell knockout mouse models were generated to elucidate the specific function of BRD4 in β cells in vivo.

Activating transcription factor 5 (ATF5) is a member of the ATF/cAMP response element binding protein (CREB) family.^[^ ^13^ ^]^ ATF5 is abundant in islets and can regulate β‐cell survival during stress.^[^ ^14^ ^]^ In different mouse models of genetic and diet‐induced obesity and diabetes, ATF5 was found to be an important regulator of β‐cell apoptosis.^[^ ^15^ ^]^ ATF5 is reportedly a downstream target of the pancreatic duodenal homeobox 1 (PDX1) pathway, which mediates β‐cell susceptibility to endoplasmic reticulum (ER) stress.^[^ ^14^ ^]^ However, whether other upstream signaling pathways can regulate ATF5 in β cells is unclear. In this study, we found that BRD4 can regulate ATF5 expression directly.

Two isoforms are encoded by the *BRD4* gene. The short form of BRD4 contains two BDs and an extraterminal (ET) region (BRD4S), and the longer form has an extended, long unique C‐terminal domain (BRDL).^[^ ^16^ ^]^ BRD4 isoforms tend to have opposite functions in tumors. BRD4S exhibits oncogenic properties, and BRD4L has a tumor suppressor role.^[^ ^17^, ^18^ ^]^ These findings suggest that the C‐terminus of BRD4L plays specific and important roles and mediates the distinct functions of BRD4L and BRD4S. Whether there are other isoforms of BRD4 in β cells is not known. BRD4 mutations, such as p.Y430C^[^ ^19^ ^]^ and p.H304Y,^[^ ^20^ ^]^ have been reported. These missense mutations are mapped to BD2 or the region close to BD2 and have strong effects on downstream targets of the BRD4 pathway, resulting in Cornelia de Lange–like syndrome or autosomal dominant syndromic congenital cataracts. However, whether BRD4 mutations are relevant to diabetes development is not known. In this study, 222 young diabetes patients were recruited, and whole exome sequencing (WES) was performed to screen for BRD4 mutations. We found that p.R749C could significantly affect BRD4 downstream signaling and might contribute to β cell malfunction. Taken together, these data indicate that BRD4‐mediated regulatory networks play crucial roles in regulating β cell differentiation and that targeting BRD4 signaling with more carefully designed chemicals may hold promise for developing novel therapeutic strategies for diabetes.

## 2 Results

### 2.1 *Brd4* Expression is Related to the β‐Cell Differentiation in a Caloric Restriction Mouse Model

**Figure 1:**
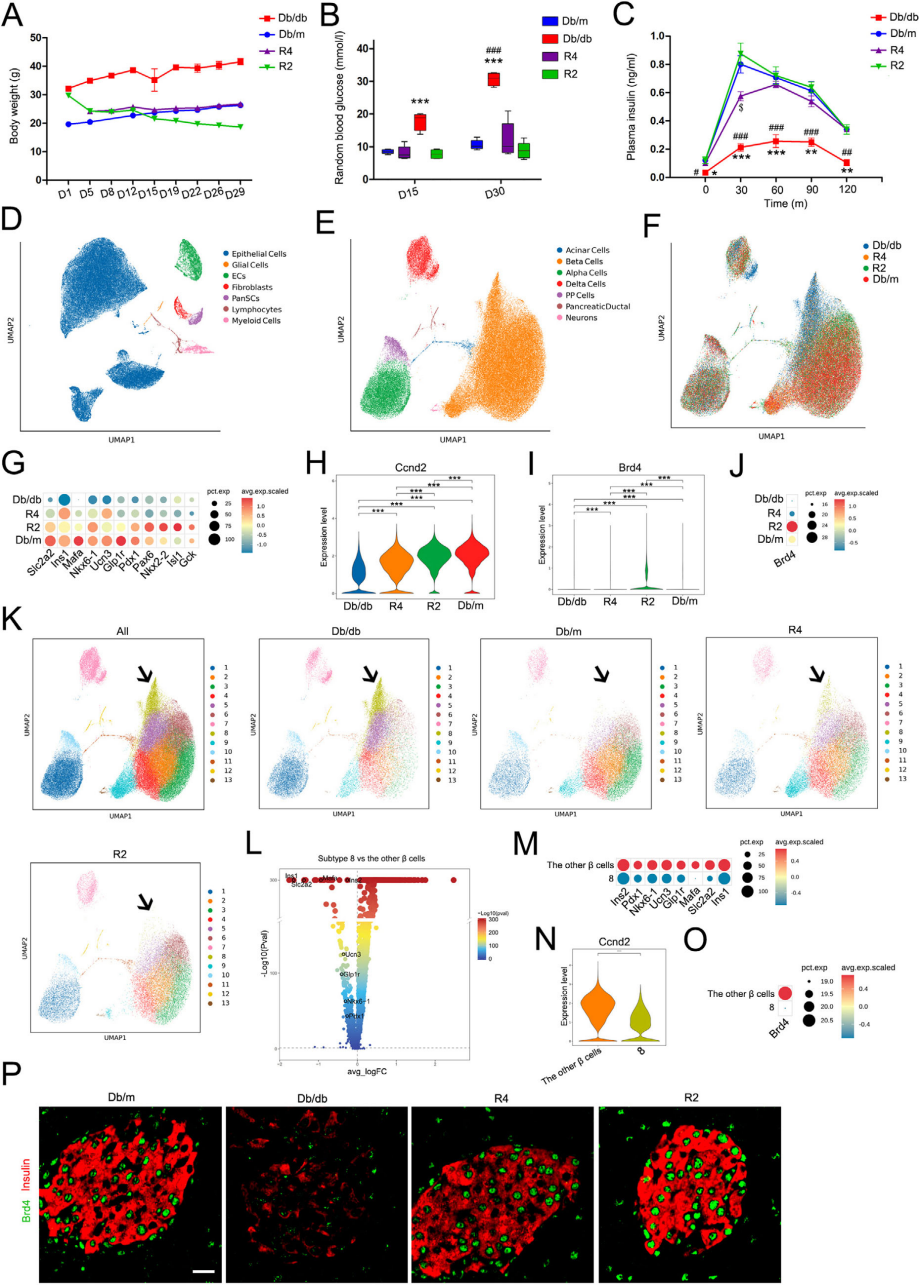
*Brd4* expression is correlated with the differentiation and function of β cells in a caloric restriction mouse model. Male db/db mice were treated with ≈50% (R4, *n* = 5) or ≈75% (R2, *n* = 5) dietary restriction, and body weights were plotted for up to 29 days in the different groups (A). Random blood glucose levels were monitored in the different groups (B). Plasma insulin levels were measured via the glucose tolerance test (IPGTT) (C). Uniform manifold approximation and projection (UMAP) representation of islet cells from db/db, db/m, R4 and R2 mice (D). Reclustering of epithelial cells into acinar cells, beta cells, alpha cells, delta cells, PP cells, pancreatic ductal cells, and neuron cells; each dot corresponds to a single cell (E). UMAP analysis of sample types of origin (F). Bubble diagram illustrating the expression of *Slc2a2*, *Ins1*, *Mafa*, *Nkx6‐1*, *Ucn3*, *Glp1r*, *Pdx1*, *Pax6*, *Nkx2‐2*, *Isl1* and *Gck* in db/db, db/m, R4 and R2 β cells. The color depth and size of each bubble are positively correlated with gene expression (G). Volcano plot showing *Ccnd2* expression in db/db, db/m, R4 and R2 β cells (H). Volcano plot demonstrating *Brd4* expression in db/db, db/m, R4 and R2 β cells (I). Bubble diagram illustrating *Brd4* expression in db/db, db/m, R4 and R2 β cells. The color depth and size of each bubble are positively correlated with *Brd4* expression (J). Reclustering of β cells into 13 subtypes represented by a UMAP plot. The arrow shows subtype 8 cells in db/db, db/m, R4 and R2 mice (K). Volcano plot (L) and bubble diagram (M) illustrating the expression of *Slc2a2*, *Ins1*, *Ins2, Mafa*, *Nkx6‐1*, *Ucn3*, *Glp1r* and *Pdx1* in subtype 8 and other β cells. Volcano plot showing *Ccnd2* expression in subtype 8 and other β cells (N). Bubble diagram illustrating *Brd4* expression in subtype 8 and other β cells. The color depth and size of each bubble are positively correlated with *Brd4* expression (O). Representative images of immunofluorescence staining for Brd4 (green) and insulin (red) in db/db, db/m, R4 and R2 islets (P). *, R4 vs db/db; #, R2 vs db/db; $, R4 vs db/m. *, # or $, *p* < 0.05; ** or ##, *p* < 0.01; *** or ###, *p* < 0.001.

It has been reported that calorie restriction (CR) could affect β‐cell differentiation in db/db mice.^[^ ^21^ ^]^ Male db/db mice were treated with ≈50% (4 g day^−1^, R4) or ≈75% (2 g day^−1^, R2) dietary restriction for 30 days. The body weight and random blood glucose levels were significantly reduced in the R4 and R2 mice (**Figure** **1**A,B). The intraperitoneal glucose tolerance test (IPGTT) revealed that blood insulin levels were significantly increased at different time points in the R4 and R2 mice compared with those in db/db mice. Moreover, blood insulin levels in the R2 mice were not significantly different from those in db/m mice but were significantly lower in the R4 mice (Figure 1C), suggesting that increased CR can further improve β‐cell function. Immunostaining revealed that Glut2, Mafa, and Pdx1 expression was significantly greater in the R4 and R2 mice than in db/db mice (Figure S1A, Supporting Information). Immunostaining also revealed that the number of Ki67‐positive β cells was significantly greater in the R4 and R2 mice than in the db/db mice (Figure S1B, Supporting Information). To explore the underlying pathways that regulate β‐cell differentiation and function, islets from the four groups were subjected to single‐cell RNA‐Seq (scRNA‐seq). A total of 104 937 cells were clustered by uniform manifold approximation and projection (UMAP) and were identified as epithelial cells and other six cell types by specific cell type markers (Figure 1D; Figure S2A, Supporting Information). Epithelial cells were reclustered and identified as acinar cells, β cells (Beta), α cells (Alpha), δ cells (Delta), PP cells, pancreatic ductal cells, and neurons by specific cell type markers via UMAP (Figure 1E; Figure S2B, Supporting Information). The sample type of origin was determined via UMAP (Figure 1F), and significantly differentially expressed genes (DEGs) between R2 and db/db β cells were identified (Figure S2C, Supporting Information). Differentiation and functional markers, such as *Slc2a2*, *Ins1*, *Mafa*, *Nkx6‐1*, *Ucn3*, *Pdx1*, *Pax6*, *Nkx2‐2*, *Isl1* and *Gck*, were significantly upregulated in the R2 mice compared with those in the db/db mice (Figure 1G). *Ccnd2* expression was significantly greater in the R2 and R4 mice than in the db/db mice (Figure 1H). *Brd4* expression was significantly greater in the R2 mice than in the db/db mice (Figure 1I,J). The automated reclustering of epithelial cells into 13 subtypes provided a comprehensive view of the islet‐cell landscape and revealed that subtype 8 β cells almost disappeared in the R2 mice compared with the db/db mice (Figure 1K; Figure S2D, Supporting Information). Differentiation and functional markers, such as *Ins1*, *Ins2*, *Mafa*, *Nkx6‐1*, *Ucn3*, *Pdx1*, and *Slc2a2*, were significantly downregulated in subtype 8 β cells compared with the other β cells (Figure 1L,M; Figure S2E, Supporting Information), which suggested that subtype eight were dedifferentiated β cells. Moreover, the expression of *Ccnd2* was significantly reduced in subtype eight cells (Figure 1N), suggesting the impairment in their proliferation capability. *Brd4* expression was barely detectable in subtype eight cells (Figure 1O), which suggests that *Brd4* expression is related to the differentiation state of β cells. Immunostaining revealed that Brd4 expression was significantly greater in R2 β cells than in db/db β cells (Figure 1P).

### 2.2 *BRD4* was Downregulated in Diabetic and Prediabetic Human β Cells

**Figure 2:**
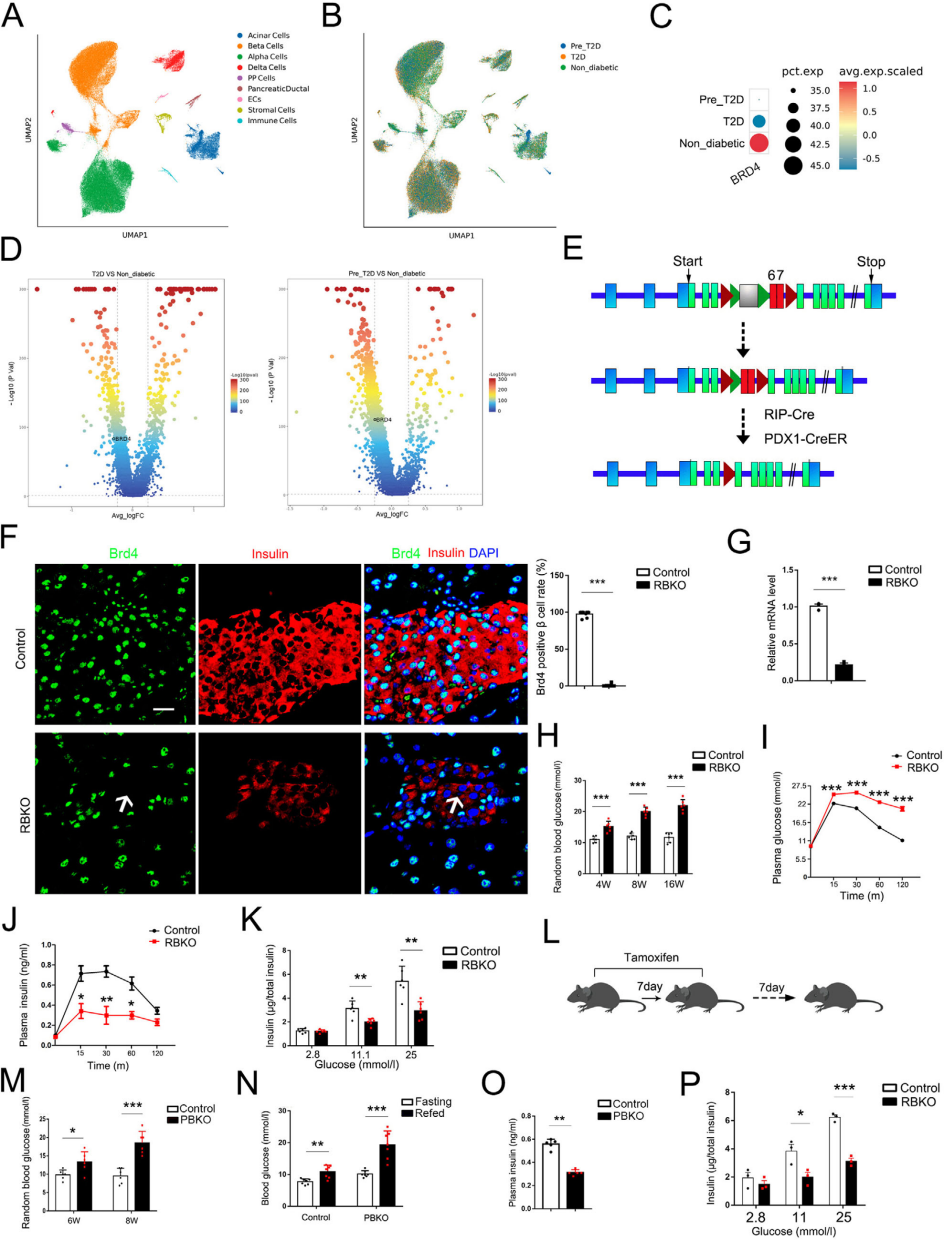
*BRD4* expression is reduced in prediabetic (Pre_T2D) and T2D β cells and *Brd4* knockout results in impaired β cell function. UMAP analysis of samples from nondiabetic, Pre_T2D and T2D (Type 2 diabetes) islets are colored by cell type (A), including acinar cells, beta cells (β cells), alpha cells, delta cells, PP cells, pancreatic ductal cells, ECS, stromal cells and immune cells, and stained by group source (B). Bubble diagram illustrates *BRD4* expression in Pre_T2D, nondiabetic and T2D β cells. The color depth and size of each bubble are positively correlated with *BRD4* expression (C). Volcano plot depicting *BRD4* expression in Pre_T2D, nondiabetic and T2D β cells. The data are colored according to expression level, and the legend is labeled on a log scale (D). Schematic diagram of the *Brd4* ^‐/‐^ mouse model construction and treatment process. RIP‐*Cre* ^+^/*Brd4* ^flox/flox^ (RBKO) or Pdx1‐*CreER* ^+^/*Brd4* ^flox/flox^ (PBKO) mice were generated using the Cre‐LoxP recombination system. Exons 6 and 7 are deleted upon *Brd4*‐*Cre*‐mediated recombination (E). Representative immunofluorescence (IF) images of Brd4 (green) and insulin (red) in pancreatic islets from control and RBKO mice. The arrow shows Brd4 knockout β cells (*n* = 7, F). qPCR analysis of *Brd4* gene expression in control and RBKO mice (*n* = 3, G). Plasma glucose levels were measured in randomly selected control and RBKO mice at 4, 8, and 16 weeks (*n* = 6, H). The results of the intraperitoneal glucose tolerance test (IPGTT) of the control and RBKO mice (I). Plasma insulin levels during the IPGTT in the control and RBKO mice (J). Glucose‐stimulated insulin secretion (GSIS) experiments revealed effects of low (2.8 mm), moderate (11.1 mm) and high (25 mm) glucose concentrations on insulin secretion (µg/total insulin) in the islets of control and RBKO mice (*n* = 6, K). Schematic model of tamoxifen‐induced *Cre*‐dependent deletion of the conditional *Brd4* alleles in the Pdx1‐*CreER*/*Brd4* ^flox/flox^ (PBKO) mice (L). Plasma glucose levels were randomly measured in control and RBKO mice at 6 and 8 weeks of age (*n* = 6, M). Fasting and refed blood glucose levels in control and RBKO mice (*n* = 7, N). Plasma insulin levels were measured in control and PBKO mice (*n* = 5, O). GSIS experiments revealed effects of low (2.8 mm), moderate (11.1 mm) and high (25 mm) glucose concentrations on insulin secretion (µg/total insulin) in the islets of control and PBKO mice (*n* = 3, P). The values are expressed as the means ± SEM. *p < 0.05; **p < 0.01; ***p < 0.001. Scale bar = 20 µm.

To investigate BRD4 function in human β cells, single‐nuclear RNA sequencing (snRNA‐seq) data from nondiabetic, prediabetic, and type 2 diabetes (T2D) islets were re‐analyzed.^[^ ^22^ ^]^ A total of 121 310 nuclei from the three groups were clustered by UMAP, and different cells were identified by cell type markers (**Figure** **2**A,B; Figure S3A,B, Supporting Information). Significantly DEGs between prediabetic and nondiabetic β cells and between T2D and nondiabetic β cells were identified (Figure S3C,D, Supporting Information). At the single‐cell level, *BRD4* was widely expressed in most islet cells (Figure S3E,F, Supporting Information). β cells were reclustered by UMAP, and *BRD4* was significantly reduced in prediabetic, and T2D β cells showed in the bubble diagram (Figure 2C) and the volcano plot (Figure 2D), suggesting that BRD4 expression in β cells was related with T2D progression.

### 2.3 β‐Cell Function was Impaired After *Brd4* Knockout

To explore BRD4 function in β cells, we constructed two *Brd4* conditional knockout mouse models. Using the Cre‐LoxP recombination system, RIP‐*Cre* ^+^/*Brd4* ^flox^/^flox^ mice (RBKO) or Pdx1‐*CreER* ^+^ */Brd4* ^flox^/^flox^ mice (PBKO) were generated. Notably, deletions of exons 6 and 7 occurred upon Cre‐mediated recombination in *Brd4* (Figure 2E). At the cellular level, immunofluorescence (IF) staining revealed a reduction in Brd4 protein levels in the RBKO β cells (Figure 2F). PCR analysis further confirmed a decrease in *Brd4* mRNA levels in the RBKO mice (Figure 2G). Random blood glucose levels in RBKO mice at 4, 8, and 16 weeks were significantly increased (Figure 2H). According to the results of IPGTT, blood glucose levels were significantly increased at different time points in the RBKO mice than in the control mice (Figure 2I), and plasma insulin levels were significantly lower (Figure 2J). Glucose‐stimulated insulin secretion (GSIS) experiments also showed that RBKO islets secreted significantly less insulin at higher glucose concentrations than control islets (Figure 2K). These findings further support a decreased insulin secretion phenotype in RBKO mice and emphasize the central role of BRD4 in regulating pancreatic β‐cell function.

To simulate the effects of BRD4 inhibitor treatment, we also generated an acute or short‐term *Brd4* conditional knockout (PBKO) model, which was generated by breeding tamoxifen‐induced *Cre* mice with *Brd4* flox mice. In this study, Pdx1‐*CreER* was used. Tamoxifen was injected intraperitoneally once every 24 h for a total of 7 consecutive days to induce recombination, and the phenotype was observed on day 14 (Figure 2L). IF revealed that Brd4 was knocked out in both β (insulin‐positive) and δ (somatostatin‐positive) cells but not in glucagon‐positive cells (Figure S4A, Supporting Information). Accordingly, IF showed that Cre signals could be detected in β and δ cells but not in α cells (Figure S4B, Supporting Information), which explained why *Brd4* was only deleted in β and δ cells. Generally, the most impressive phenotypic change in PBKO mice was an increase in blood glucose levels, including random, fasting, and refed blood glucose levels (Figure 2M,N). Plasma insulin levels were significantly lower in PBKO mice than in control mice (Figure 2O). GSIS experiments showed that PBKO islets secreted significantly less insulin at higher glucose concentrations than control islets (Figure 2P), which suggested that β cell function was impaired after acute deletion of *Brd4*.

**Figure 3:**
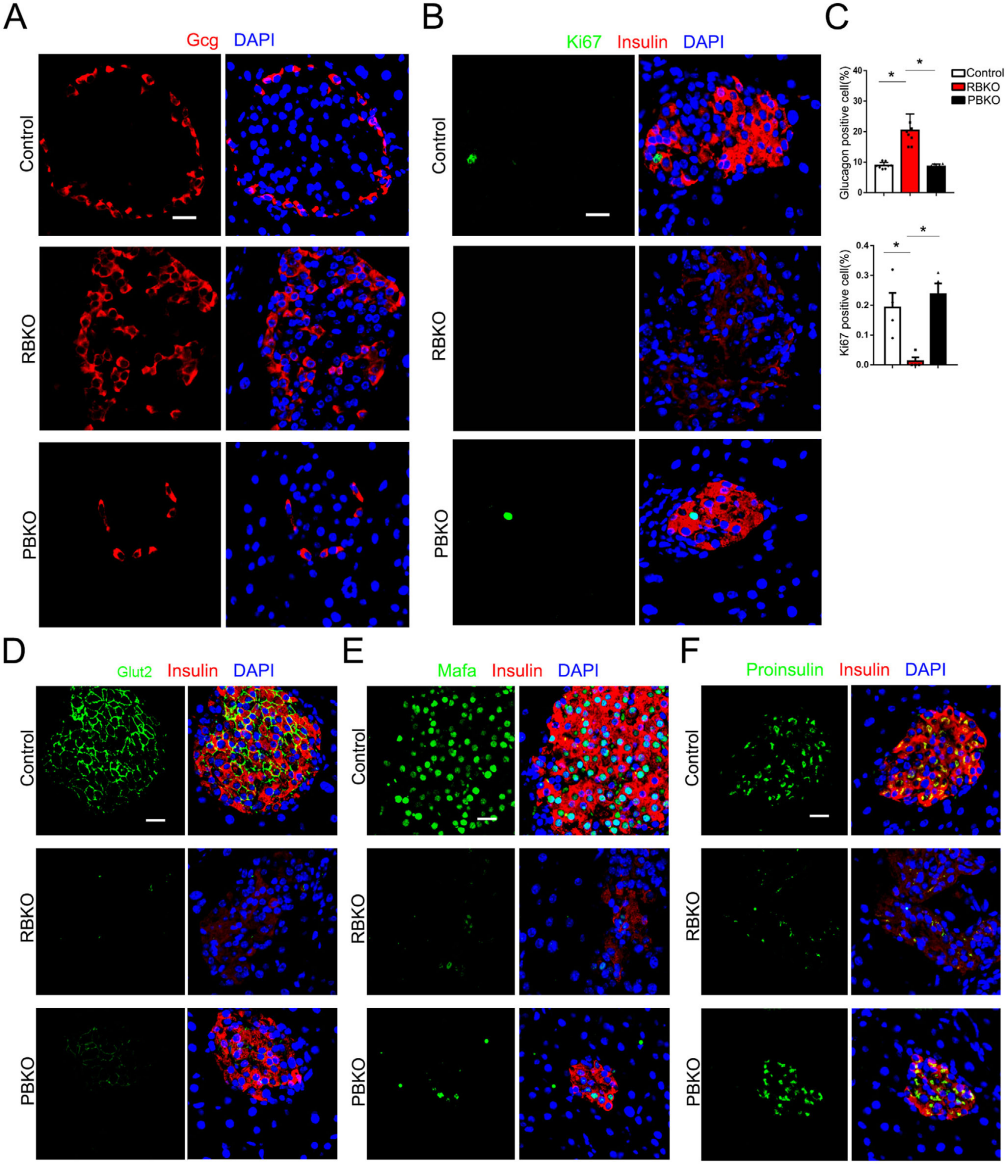
*Brd4* deletion impairs β‐cell differentiation and proliferation. Representative IF images depicting Gcg (red) and DAPI (blue) staining in pancreatic islets from RBKO, PBKO, and control mice (*n* = 8, A). Representative IF images depicting Ki67 (green), insulin (red), and DAPI (blue) staining in pancreatic islets from RBKO, PBKO, and control mice (*n* = 4, B). Quantitative analysis of the percentages (%) of Gcg‐positive cells and Ki67‐positive cells revealed by IF analysis of Figure A,B (C). Representative IF images depicting Glut2 (green, D), Mafa (green, E), proinsulin (green, F), and insulin (red), and DAPI (blue), in pancreatic islets from control, RBKO and PBKO mice. PBKO, Pdx1‐CreER;*Brd4* ^flox/flox^. RBKO, RIP‐Cre;*Brd4* ^flox/flox^. *p < 0.05. Gcg, Glucagon. Scale bar = 20 µm.

The α/β cell ratio in RBKO mice was altered, and the number of glucagon (Gcg)‐positive cells was significantly increased (**Figure** **3**A,C). There were significantly fewer Ki67‐positive β cells in the RBKO mice than in the PBKO and control mice (Figure 3B,C), suggesting that long‐term deletion of Brd4 results in a reduction in the proliferation of β cells. IF showed that Glut2 and Mafa expression was significantly lower in both RBKO and PBKO mice than in control mice (Figure 3D,E), suggesting that BRD4 plays important roles in β‐cell differentiation and maturation. Interestingly, proinsulin levels were significantly lower in RBKO β cells than in PBKO and control β cells (Figure 3F), suggesting that insulin synthesis rather than processing is impaired after long‐term deletion of *Brd4* in β cells. Taken together, our data show that long‐term deletion of *Brd4* has an impact on β‐cell proliferation and differentiation, and acute deletion mainly affects differentiation and maturation.

### 2.4 ScRNA‐Seq Revealed that *Brd4* Deficiency Influences β‐Cell Differentiation

**Figure 4:**
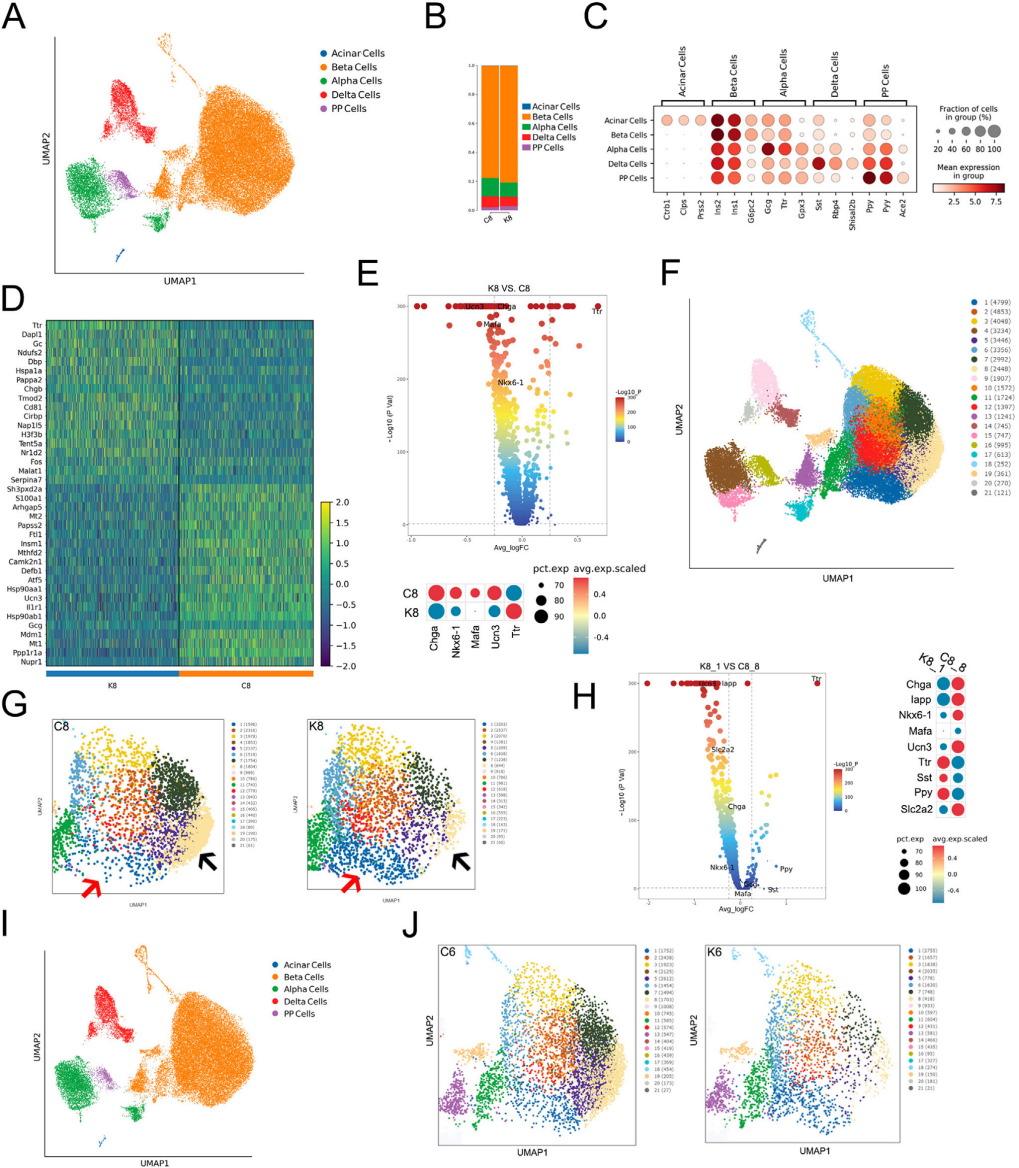
ScRNA‐seq revealed dynamic cellular subtypes and gene expression changes in *Brd4*‐deficient β cells. UMAP analysis of islets from 8‐week‐old control (C8) and PBKO (K8) mice stratified by cell type, including acinar cells, beta cells (β cells), alpha cells, delta cells, and PP cells; each dot corresponds to a single cell (A). The mean proportion of each cell subtype is presented for the C8 and K8 groups (B). Bubble chart showing the expression of the top marker genes in each cell cluster (C). Heatmap of the top 20 significant DEGs whose expression increased or decreased in the C8 and K8 β cells. The data are colored according to expression level, and the legend is labeled on a log scale (D). Volcano plot and bubble diagram depicting significant DEGs related to β‐cell differentiation and function between K8 and C8. The data are colored according to expression level, and the legend is labeled on a log scale (E). Reclustering of islet cells into 21 subtypes represented by a UMAP plot (F). β‐cell subtypes identified in the UMAP plot for C8 and K8. The arrow shows the changes in subtypes between C8 and K8 β cells (G). Volcano plot and bubble diagram depicting significant DEGs related to β‐cell maturation and differentiation between subtype 1 of K8 (K8_1) and subtype 8 of C8 (C8_8) β cells. The data are colored according to expression level, and the legend is labeled on a log scale (H). UMAP dimension‐reduction projection analysis of islets from control (C6) and PBKO (K6) mice at 6 weeks of age by cell type, including acinar cells, beta cells (β cells), alpha cells, delta cells, and PP cells; each dot corresponds to a single cell (I). β cell subtypes identified in the UMAP plot with C6 and K6 (J). PBKO, Pdx1‐CreER;*Brd4* ^flox/flox^.

To explore the roles that Brd4 plays in β cells and to reduce the secondary effects of long‐term hyperglycemia, PBKO islets were subjected to scRNA‐seq. A total of 41 121 cells from PBKO and control mice at the age of 8 weeks (K8 and C8) were clustered by UMAP, and they were identified as acinar cells, β cells (Beta), α cells (Alpha), δ cells (Delta) and PP cells by specific cell‐type markers (**Figure** **4**A–C; Figure S5A–C, Supporting Information). Since the main phenotypic change in PBKO mice is a reduction in insulin secretion, we focused on β cells. The top significantly DEGs in the C8 and K8 β cells were shown in a heatmap (Figure 4D). At single‐cell resolution, the expression of differentiation markers such as *Chga*, *Nkx6‐1*, *Mafa*, and *Ucn3* was significantly reduced after *Brd4* knockout, and the expression of transthyretin (*Ttr*), which is specifically expressed in α cells,^[^ ^23^ ^]^ was significantly increased in PBKO β cells (Figure 4E). These findings suggest that β‐cell differentiation is impaired after acute *Brd4* deletion. Subsequently, the automated reclustering of islet cells into 21 subtypes, represented by a UMAP plot, provided a comprehensive view of the β‐cell landscape (Figure 4F). Distinguishing β cell subtypes within the UMAP plot revealed notable variations between the C8 and K8 groups, with an increase observed in subcluster 1 and a decrease in subcluster 8 in K8 compared with C8 (Figure 4G). DEG analysis revealed that β‐cell‐specific differentiation markers, such as *Chga*, *Nkx6‐1*, *Mafa*, *Ucn3*, and *Slc2a2*, were significantly reduced in subcluster 1 in K8, and other islet cell markers, such as *Sst*, *Ppy*, and *Ttr*, were significantly increased (Figure 4H). These findings suggest that more PBKO β cells obtained a “progenitor‐like” expression profile. We also validated these findings in younger PBKO and control mice at 6 weeks of age (K6 and C6). A total of 38,404 cells from K6 and C6 were clustered according to UMAP (Figure 4I; Figure S5D, Supporting Information). DEG analysis revealed that *Chga*, *Ucn3*, and *Slc2a2* were significantly reduced in K6 β cells (Figure S5E,F, Supporting Information), which was also observed in K8 β cells. Moreover, 21 subtypes were also clustered in the K6 and C6 islet cell populations, similar to the K8 and C6 islet cell populations (Figure S5G, Supporting Information). Subcluster 1 was significantly increased and subcluster 8 was reduced in K6 β cells compared with in C6 β cells (Figure 4J), which was consistent with the findings in older PBKO mice. Overall, our study showed that after acute *Brd4* knockout, the differentiated state cannot be maintained in β cells.

### 2.5 ScATAC‐Seq Reveals β Cell Transcription Factor Activity and Chromatin Interaction Networks in *Brd4* Deficiency

**Figure 5:**
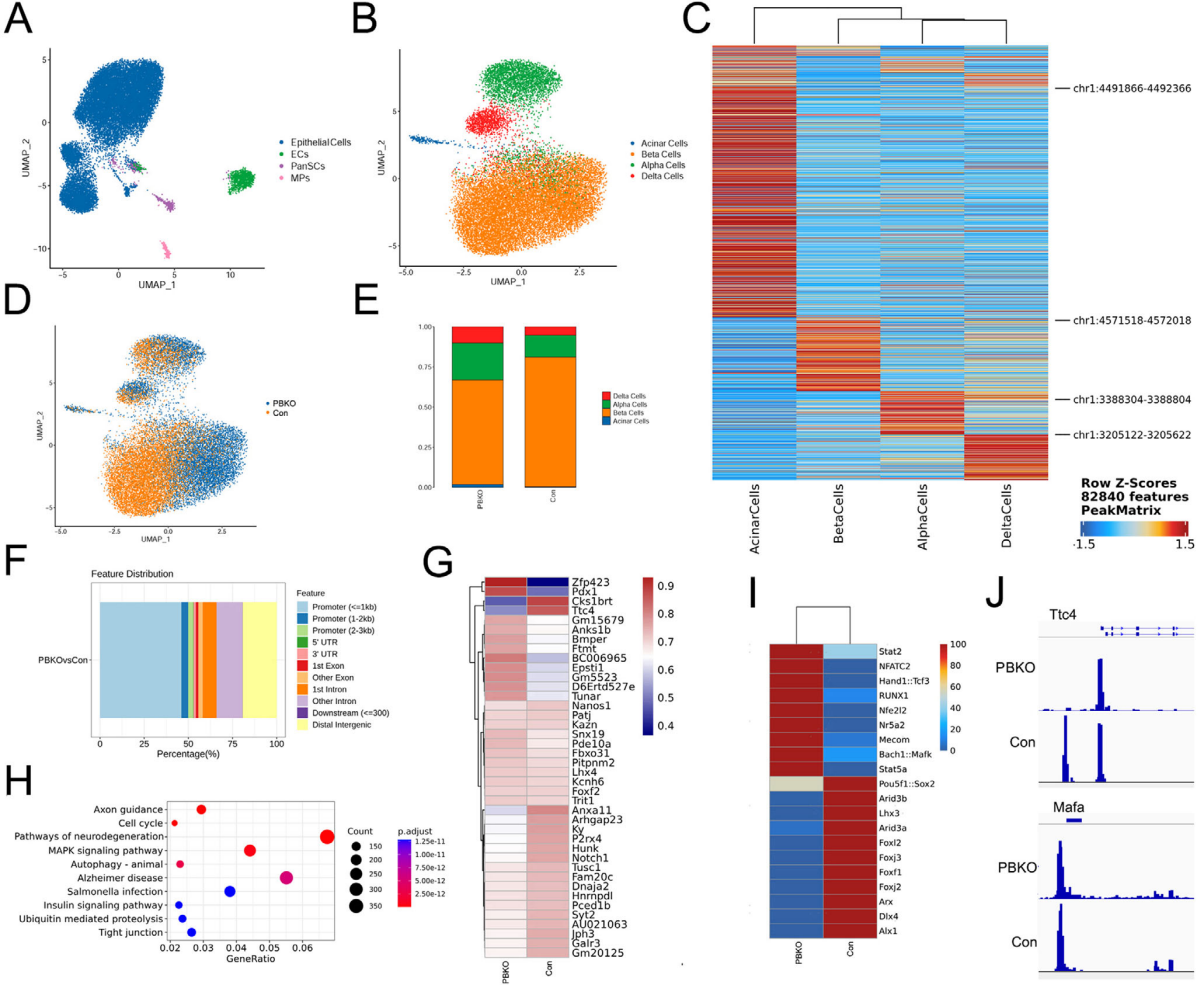
ScATAC‐seq revealed that chromatin accessibility was altered in *Brd4*‐deficient β cells. Analysis of chromatin accessibility patterns within transcription factor start sites (TFSSs) revealed the distinct grouping of islet cells into PanSCs, MCs, ECs, and epithelial cells, and each dot corresponds to a single cell (A). Clustering of distinct epithelial cells, including alpha cells, beta cells (β cells), acinar cells, and delta cells, based on chromatin accessibility patterns within the TFSS (B). Heatmap of annotated characteristic peaks for chromatin accessibility in different cell types (C). UMAP visualizations of control and PBKO islet cells based on chromatin accessibility patterns within the TFSS (D). Proportion of PBKO and control epithelial cells (E). Bar plot of the annotated differential peaks for chromatin accessibility between PBKO and control β cells; the main difference was located in the promoter region (F). Heatmap of the top 20 DEGs in PBKO and control β cells according to the scATAC gene score (see Experimental Section) (G). GO enrichment analysis of DEGs based on the scATAC gene score (H). Heatmap of the top differentially expressed transcription factors in PBKO and control β cells based on transcription factor activity analysis (see Experimental Section) (I). IGV visualization of peaks representing chromatin accessibility in the *Ttc4* and *Mafa* promoter regions in PBKO and control β cells (J). Con, control. PanSCs, pancreatic stellate cells. MCs, mesothelial cells. ECs, endothelial cells. PBKO, Pdx1‐CreER;*Brd4* ^flox/flox^.

Since Brd4 is an essential epigenetic regulator, we also applied scATAC‐seq to PBKO and control islets to determine whether the chromatin landscape and epigenetic features changed after *Brd4* was knocked out in β cells. A total of 22 448 islet cells, most of which were identified as epithelial cells, were clustered by UMAP based on the chromatin accessibility of the cell‐type marker genes (**Figure** **5**A; Figure S6A,B, Supporting Information). Furthermore, the UMAP projection revealed acinar cells, β cells (Beta), α cells (Alpha) and δ cells (Delta) (Figure 5B) according to the chromatin accessibility of specific cell type marker genes (Figure 5C). Notably, the cluster dots representing PBKO β cells shifted rightward (Figure 5D), suggesting that the chromatin landscape significantly changes after *Brd4* deletion. The UMAP projection also showed that the number of β cells was clearly reduced (Figure 5E) in the PBKO mice, as indicated by the scATAC‐seq data. The locations of chromatin accessibility peaks were significantly different between PBKO and control β cells, and the differences were mainly in the promoter regions of the target genes (Figure 5F; Figure S6C, Supporting Information). DEGs based on the scATAC gene score (see Experimental Section) were identified (Figure 5G; Figure S6D, Supporting Information), and functional enrichment analysis revealed that these DEGs could affect the cell cycle, neurodegeneration, autophagy, and insulin signaling pathways (Figure 5H). Differentially expressed transcription factors (DTFs) were also analyzed based on transcription factor activity analysis (see Experimental Section; Figure 5I), and these DTFs influenced many important pathways, including cell fate commitment signaling (Figure S6E, Supporting Information). In particular, the number and height of chromatin‐accessibility peaks was significantly lower in the promoter regions of *Ttc4* and *Mafa* (Figure 5J). Ttc4 and its other family members were found to play important roles in the cell cycle and transcriptional regulation and to be relevant to type 2 diabetes development.^[^ ^24^ ^]^ Therefore, chromatin remodeling and gene accessibility were evidently affected after acute *Brd4* knockout in β cells, which highlights the important roles that Brd4 plays in β cells

### 2.6 BRD4 Knockdown in Human Islet Organoids Results in β Cell Malfunction

**Figure 6:**
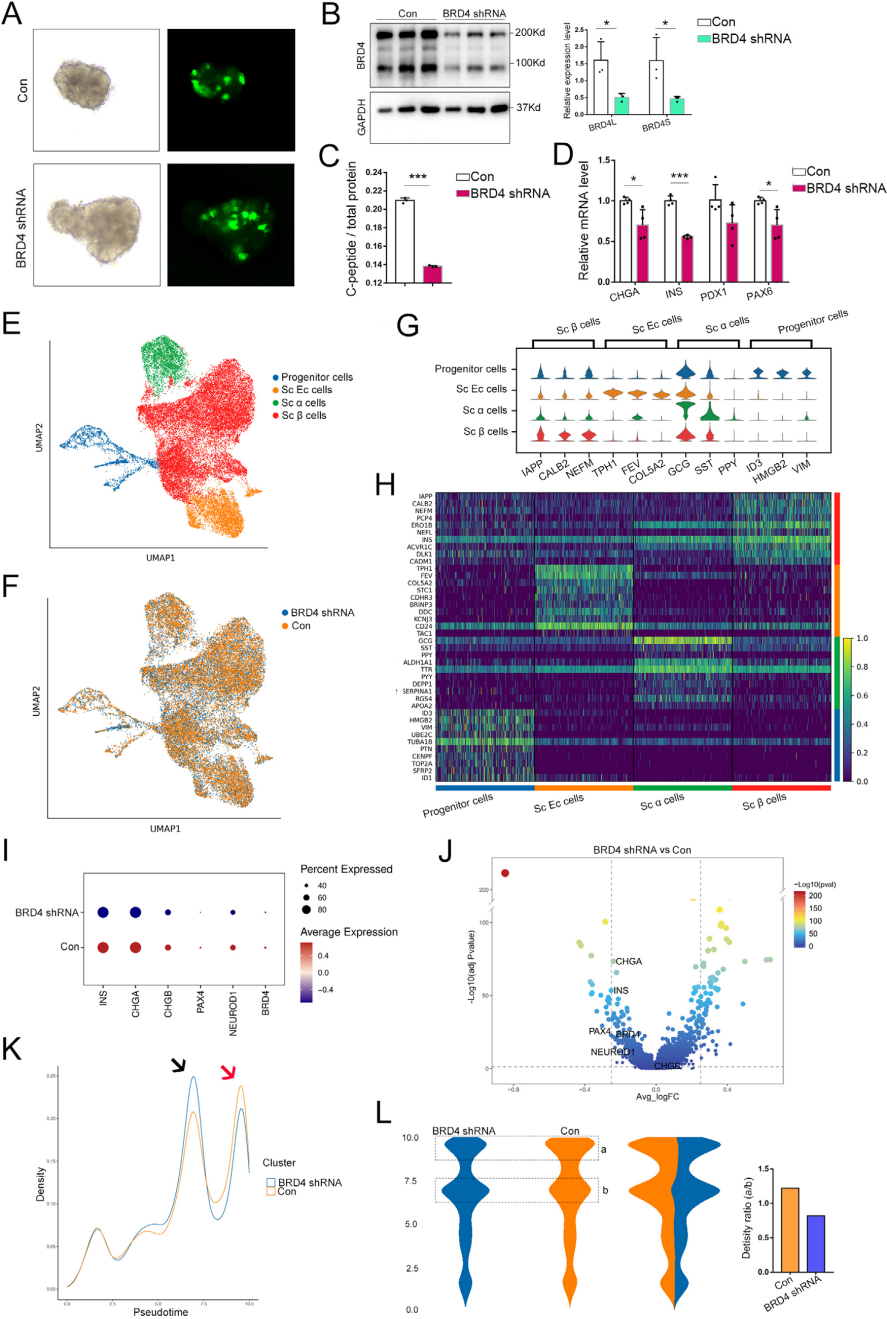
BRD4 knockdown impair β cell function in human islet organoids. Representative images showed the infection of *BRD4*‐shRNA lentivirus in human islet organoids. GFP expression was driven by a CMV promoter and used to track the infection efficiency (green) (A). BRD4 knockdown was validated by western blotting. Both BRD4L (long isoform) and BRD4S (short isoform) can be detected, and GAPDH was used as an internal control (*n* = 3, B). C‐peptide in whole islet organoids was measured by ELISA, and normalized by total protein (*n* = 3, C). qPCR showed mRNA level of *CHGA*, *INS*, *PDX1* and *PAX6* after BRD4 knockdown in whole islet organoids (*n* = 4, D). UMAP analysis of cells from control (Con) and BRD4‐knockdown islet organoids stratified by cell type, including human pluripotent stem cell‐derived (SC) β cells, progenitor cells, SC α cells and SC EC (enterochromaffin) cells; each dot corresponds to a single cell (E). UMAP visualization of the single‐cell transcriptomic dataset of islet organoids cells. Different colors represent different samples (F). Violin plot of cell type marker genes that identified the clusters generated by UMAP plotting (G). Heatmap of the top 20 significant DEGs in different cell type (H). Bubble diagram illustrating the expression of *INS, CHGA, CHGB, PAX4, NEUROD1*, and *BRD4* in control and BRD4‐knockdown SC β cells. The color depth and size of each bubble are positively correlated with gene expression (I). Volcano plot showing *INS, CHGA, CHGB, PAX4, NEUROD1*, and *BRD4* in control and BRD4‐knockdown SC β cells (J). Single‐cell trajectory analysis with Monocle 2 identifies two density peaks during SC β cells differentiation, and BRD4 knockdown can affect the peak height; Different density peaks were marked by arrows with different colors (K). Monocle trajectory analysis showing the change of stream density in control and BRD4‐knockdown SC β cells; Density ratio = a/b (L). *p < 0.05; ***p < 0.001.

In this study, BRD4 was knocked down in islet organoids to validate its function in human β cell. Islet organoids were infected via lentiviral *BRD4* shRNA with a GFP as an infection tracking marker (**Figure** **6**A; Figure S6F,G, Supporting Information). BRD4 knockdown was validated by western blotting, and both full‐length (long isoform, BRD4L) and short isoform (BRD4S) can be significantly repressed by ≈66% (Figure 6B). C‐peptide content was significantly reduced after BRD4 knockdown in whole islet organoids (Figure 6C). qPCR showed that *INS*, *CHGA* and *PAX6* were significantly reduced in BRD4‐knockdown organoids (Figure 6D). BRD4‐knockdown and control organoids were subjected to scRNA‐seq, and a total of 28 835 cells were clustered by UMAP (Figure 6E,F), and they were identified as progenitor cells, β cells, α cells, and EC (enterochromaffin cell) cells by specific cell‐type markers (Figure 6G).The top significantly DEGs in the control and BRD4‐knockdown cells are shown in a heatmap (Figure 6H). At single‐cell resolution, the expression of differentiation and function markers such as *CHGA*, *CHGB*, *NEUROD1*, *PAX4*, and *INS* were significantly reduced in BRD4‐knockdown β cells. *BRD4* expression was also significantly lower which suggests that the knockdown is successful (Figure 6I,J). The process of β cells differentiation in islet organoids was stimulated and reconstructed with Monocle 2, and two peaks in the differentiation trajectories were identified. After BRD4 knockdown, less β cells were located in the final differentiation peak (Figure 6K,L), which suggests lower BRD4 could impair human β cell differentiation.

### 2.7 ATF5 can be Directly Regulated by BRD4

**Figure 7:**
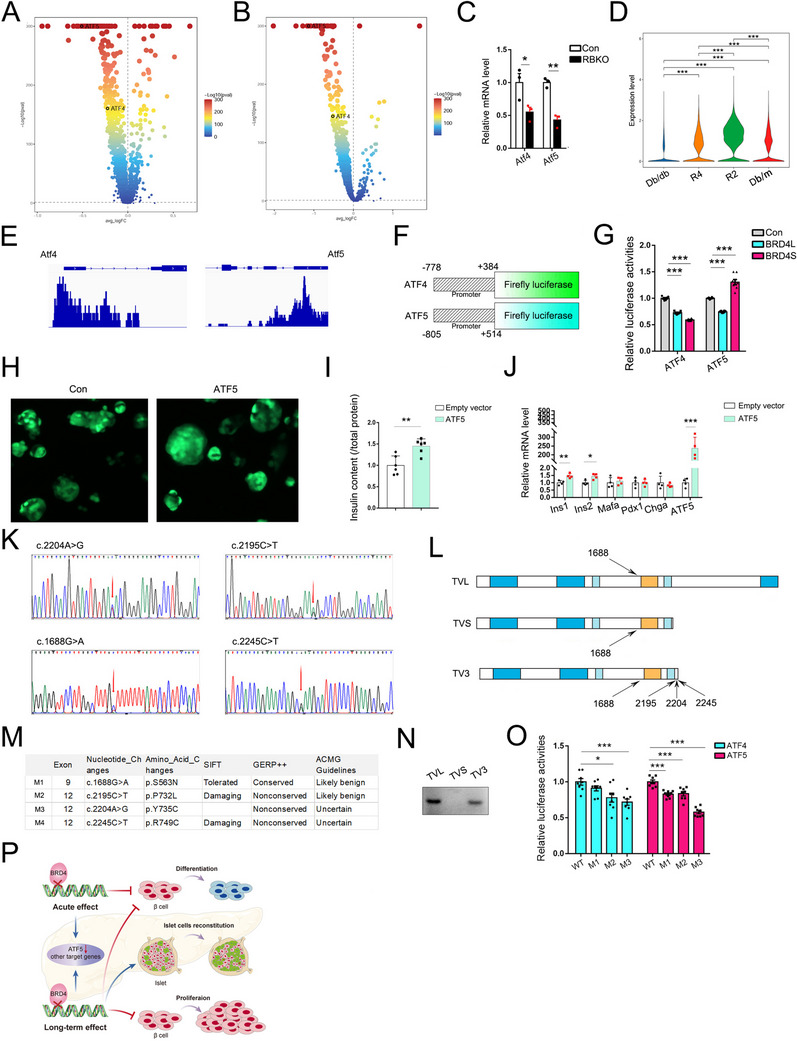
ATF5 is a target of the BRD4 pathway, and the p.R749C mutation can affect BRD4‐ATF5 signaling. Volcano plot depicting *Atf4*/*Atf5* changes between K8 and C8 β cells. The data are colored according to expression level, and the legend is labeled on a logarithmic scale (A). Volcano plot depicting significant changes in *Atf4*/*Atf5* expression between K8_1‐subtype and C8_8‐subtype β cells (B). qPCR analysis of *Atf4* and *Atf5* gene expression in the islets of control and RBKO mice (*n* = 3, C). Volcano plot depicting significant changes in *Atf5* expression in db/db, db/m, R4 and R2 β cells (D). IGV visualization of Brd4 binding peaks at the promoter regions of *Atf4* and *Atf5* in INS1 cells based on ChIP‐seq data (E). A schematic diagram of the *ATF4* and *ATF5* luciferase reporters (F). *ATF4* and *ATF5* expression can be regulated by BRD4L and BRD4S, as indicated by luciferase assays. Firefly luciferase activities were normalized to Renilla luciferase activities (*n* = 7, G). Representative images showed RBKO islets were transduced by control (empty vector, GFP only) and ATF5 (ATF5‐P2A‐GFP) lentivirus. Green is GFP signal (H). Insulin content were measured after ATF5 overexpression in RBKO islets (*n* = 6, I). qPCR analysis of *Ins1, Ins2, Mafa, Pdx1, Chga* and *ATF5* expression in the RBKO islets after ATF5 overexpression (*n* = 4, J). BRD4 mutations were validated by Sanger sequencing (K). Mutations were mapped to different transcript variants of *BRD4*, and different functional domains are colored (L). The table shows basic information about BRD4 mutations and functional prediction (M). PCR detection of different *BRD4* transcript variants in human islets (N). The impact of mutations on the BRD4‐ATF4/ATF5 pathway indicated by a luciferase assay. Firefly luciferase activities were normalized to Renilla luciferase activities (*n* = 8, O). A schematic diagram of the BRD4‐ATF5 pathway and the different effects caused by long‐term and acute BRD4 deletion in β cells (P). The values are expressed as the means ± SEM. *p < 0.05; **p < 0.01; ***p < 0.001. Con, control. BRD4L, long isoform of BRD4. BRD4S, short isoform of BRD4. TVL, transcript variant for long BRD4. TVS, transcript variant for short BRD4. TV3, BRD4 transcript variant 3. SIFT, Sorting Intolerant From Tolerant. GERP, genomic evolutionary rate profiling. ACMG, the American College of Medical Genetics and Genomics. RBKO, RIP‐Cre; *Brd4* ^flox/flox^.

*Atf4* and *Atf5* were significantly lower in K8 β cells than in C8 β cells (**Figure** **7**A; Figure S7A, Supporting Information). Similarly, *Atf4* and *Atf5* were significantly lower in K8 subcluster 1 β cells than in C8 subcluster 8 β cells (Figure 7B, Figure S7B, Supporting Information). qPCR revealed that both *Atf4* and *Atf5* were significantly lower in PBKO islets than in control islets (Figure 7C). Moreover, *Atf4* and *Atf5* were significantly lower in K6 β cells than in C6 β cells (Figure S7C, Supporting Information). *Atf5* were significantly increased in R2 compared with db/db β cells (Figure 7D). Reanalysis of public ChIP‐seq data showed that there was a strong binding peak for BRD4 in the promoter regions of *Atf4* and *Atf5* both in β cell line, INS1, and non‐β cell lines, 293 and HepG2 cells (Figure 7E; Figure S7D, Supporting Information). Interestingly, we did not observe any change in chromatin accessibility in the promoter regions of *Atf4* or *Atf5* after *Brd4* knockout via scATAC‐seq (Figure S7E, Supporting Information). Since BRD4 has histone‐binding‐indepdent transcriptional functions, we generated luciferase reporters using the human promoter regions of human *ATF4* and *ATF5* to determine whether BRD4 can directly regulate ATF4 and ATF5 expression (Figure 7F). Luciferase assays revealed that two BRD4 isoforms, BRD4L and BRD4S, could significantly repress *ATF4* expression. However, BRD4L and BRD4S had opposite regulatory effects on *ATF5* expression. BRD4L reduced *ATF5* expression, while BRD4S increased *ATF5* expression, as indicated by the *ATF5* reporter (Figure 7G). Here, our study suggested that ATF4 expression is regulated by multiple potential factors that reduce ATF4 expression even after *BRD4* knockout, and BRD4 looks like an “add‐on” instead of an essential factor for ATF4 regulation. However, ATF5 is directly positively regulated by BRD4S. To validate ATF5 function in BRD4 signaling, rescue experiments were performed. Human ATF5 CDS fused with P2A‐GFP were packaged into a lentivirus. RBKO islets were transduced with ATF5‐P2A‐GFP or control lentivirus (Figure 7H). ATF5‐P2A‐GFP was driven by CMV promoter. Insulin content was significantly increased after ATF5 overexpression in RBKO islet (Figure 7). *Ins1* and *Ins2* were significantly increased after ATF5 overexpression, wherea *Mafa*, *Pdx1*, and *Chga* were not changed (Figure 7J), which suggests that restoring ATF5 in *Brd4* knockout islets can improve β‐cell function to a certain extent.

### 2.8 The p.R749C Mutation can Affect BRD4‐ATF4/ATF5 Signaling

To explore whether BRD4 mutations can contribute to diabetes development, we recruited 222 young diabetes patients whose clinical characteristics are summarized in Table S1 (Supporting Information) to screen for BRD4 mutations. Finally, four mutations, namely, c.1688G‐>A (p.S563N), c.2195C‐>T (p.P732L), c.2204A‐>G (p.Y735C) and c.2245C‐>T (p.R749C), were detected by WES and validated by Sanger sequencing (Figure 7K). c.1688G‐>A was mapped to all three transcript variants (TVL, TVS, and TV3) of BRD4, and the other three mutations were specifically mapped to TV3 (Figure 7L). TVL encodes the long isoform of BRD4 (BRD4L), and TVS encodes the best‐known short isoform of BRD4 (BRD4S) in some tumors.^[^ ^16^ ^]^ TV3, which has been poorly studied, can also be translated into a short isoform that has 72 amino acid residues longer than traditional BRD4S. Since Sorting Intolerant from Tolerant (SIFT)^[^ ^25^ ^]^ predicted that the c.1688G‐>A mutation was unlikely to have an adverse effect on BRD4 function (Figure 7M), we focused on the other three mutations. We designed primers that targeted the specific regions of the three transcript variants. PCR with different primers revealed that only TVL and TV3 were detected in human islets, as indicated by the high‐fidelity polymerase enzyme (Figure 7N; Figure S7F, Supporting Information). Wild‐type (WT) and mutated TV3 along with *Atf4* or *Atf5* reporters were co‐transfected into the INS1 cell line, and a luciferase assay showed that c.2245C‐>T (p.R749C) had the greatest effect on *Atf4* and *Atf5* expression (Figure 7O). These findings suggest that p.R749C can affect BRD4 function and downstream targets to a certain extent.

Overall, our comprehensive exploration revealed the critical role of BRD4 in β cells. BRD4 plays a fundamental role in maintaining β cell differentiation because both long‐term and acute BRD4 deficiency resulted in a reduction in insulin secretion and downregulation of differentiation markers. Our study also showed that ATF5 is a functional target in the BRD4 pathway and might be a potential target for BRD4 signaling intervention (Figure 7P).

## 3 Discussion

In this study, our investigation of Brd4 deficiency in murine models yielded crucial insights into BRD4 function in β cells. Specifically, in contrast to acute conditions, long‐term conditional *Brd4* knockout results in a reduction in β cell proliferation. This reduction might be subject to both direct effects from Brd4 deficiency during embryonic and adult stages and secondary effects from glucotoxicity due to long‐term hyperglycemia in light of the phenotypic changes in the acute knockout model. However, it is reasonable to hypothesize that differentiation or maturation effects are the direct regulatory effects of the BRD4 pathway in β cells. Functional maturity and proliferative capacity are mutually exclusive states in β cells,^[^ ^26^ ^]^ however in the RBKO model, both maturity and proliferation are impaired. RBKO β cells were not reversed to a “progenitor‐like” state; instead, they were converted to a dedifferentiated or failure state, as in the “db/db” diabetic mouse model.^[^ ^21^ ^]^ To regulate β‐cell function, more than one downstream target of the BRD4 pathway may exist. Here, we suggest that BRD4 might regulate ATF5 in an epigenetic‐independent manner, as indicated by the luciferase reporter assay, because the chromatin accessibility of the *Atf4/Atf5* promoter region did not change in the PBKO mice. Actually, BRD4 plays transcriptionally roles independent of histone binding; for example, BRD4 has been suggested to contribute to viral DNA replication which has no histone at all.^[^ ^27^, ^28^ ^]^ It was also reported that BRD4 can bind YAP/TEAD1 directly.^[^ ^29^ ^]^ Whether ATF5 is regulated by BRD4 and TEAD1 signaling interactions is an interesting topic and deserves further study since TEAD1 also plays an important role in β‐cell differentiation.^[^ ^30^ ^]^

BRD4S is mainly encoded by the best‐known transcript variant, TVS, in tumors. However, we found that TVS could not be detected in human islets, suggesting that there are cell‐ or organ‐specific expression patterns for BRD4 isoforms. Interestingly, TV3 (NCBI Reference Sequence: NM_001330384.2), which has been poorly studied and can also encode a short isoform of BRD4 (named BRD4S1 here), can be detected in human islets. Since BRD4S1 is only 72 amino acids longer than BRD4S, it is reasonable to suppose that these two short isoforms have similar functions. However, the subtle functional differences between these two short isoforms deserve further study. In young patients with diabetes, more mutations can be detected in TV3 than in TVL. In particular, the p.R749C mutation can significantly affect BRD4‐ATF4/ATF5 signaling. It is possible that p.R749C might affect the conformation of BRD4 more significantly than other missense mutations, but the details of the mechanisms involved remain unclear.

It is worth to note that in this study we used a sensitive method, luciferase assay, to evaluate p.R749C function, however, in order to draw a more solid conclusion, more methods and models, such as p.R749C mutation mouse, should be adapted to validate its function in diabetes development.

In conclusion, our study underscores the multifaceted roles that BRD4 plays in β cells. By elucidating the regulatory networks governed by BRD4 in pancreatic β cells, our study revealed that the therapeutic targeting of the BRD4 pathway represents a potent and promising strategy for treating diabetes.

## 4 Experimental Section

### Clinical Samples

Peripheral blood were collected from patients or their parents and submitted with a requisition form, which included informed consent and patient clinical data. All of the protocols were approved by the Qilu Hospital Ethics Committee, Shandong University (KYLL‐202212‐062).

### *Brd4* Conditional Knockout Mouse

*Brd4* ^flox/flox^ mice were purchased from Cyagen (https://www.cyagen.com/us/en/) and bred with mice expressing Cre recombinase driven by the rat insulin promoter (Rip‐Cre) or tamoxifen‐inducible Pdx1‐Cre strain (Jackson Laboratory, #024968) to generate the *Brd4* conditional knockout mice. Tamoxifen (75 mg kg^−1^ body weight) was administered via intraperitoneal injection (using an ACUC‐approved injection procedure) once every 24 h for a total of 7 consecutive days to induce recombination. *Brd4* ^flox/flox^ mice were genotyped using *Brd4*‐flox‐forward (TACAAGGAAGGGATGTTTTAACGC) and reverse (TGCTAACAGGTTGTATTCACAAGG) primers. Heterozygous Rip‐Cre or *Brd4* ^flox/flox^ mice were used as control. All of the mice were housed in pathogen‐free facilities with a 12h light/dark cycle and had free access to water and food. All mice used for the experiments were males. All of the animal experiments were conducted in accordance with the Guide for the Care and Use of Laboratory Animals published by the National Institutes of Health.

### Calorie Restriction Mouse Model

The C57BLKS/J‐Leprdb/Leprdb (db/db) and C57BLKS/J‐Leprdb/m (db/m) male mice were purchased from the Shanghai Laboratory Animal Center, Chinese Academy of Sciences (SLAC, CAS). All of the mice were housed in pathogen‐free facilities with a 12 h light/dark cycle. Male 5‐week‐old db/db mice were randomly assigned to an ad libitum diet with free access to regular chow (db/db‐F), or receiving limited food supply (4 g day^−1^, R4; 2 g day^−1^, R2) for 1 month. Food was added for R4 and R2 mice at fixed time every day (12 o'clock). As control, male db/m mice were also given free access to regular chow (db/m‐F) for 1 month. For R2 mice, 20% body weight loss was observed on day 15. Since the morphology of R2 islets had no impressive changes compared with that of db/db‐F indicated by HE staining on day 15, the 2 g day^−1^ food supply was extended to 29 days. All of the animal experiments were conducted in accordance with the Guide for the Care and Use of Laboratory Animals published by the National Institutes of Health. Animal use had been reviewed and approved by the Animal Ethical and Welfare Committee (AEWC) of Shandong University (DWLL‐2024‐089).

### Cell Line

INS1 cell line was cultured and maintained as previously described.^[^ ^30^ ^]^ Transfection was performed using the Lipofectamine 3000 kit (Thermo Fisher) according to the manufacturer's instructions.

### Human Islets Organoids (HIO)

HIO were generated as previously described.^[^ ^31^ ^]^ Briefly, before differentiation, adherent hCiPSCs (at 80–90% confluence) were dispersed into single cells using Accutase (EMD Millipore, #SCR005), rinsed with DMEM/F12 (Gibco, #11330‐032) and seeded on Matrigel‐coated cell factory (Thermo Fisher Scientific, #169171) at ≈1.35 × 10^5^ cells per cm^2^ in mTESR1 supplemented with 10 µm Y27632. Differentiation was initiated 24 h after seeding by using different small molecules and cytokines in the six stages differentiation process.

### Whole Exome Sequencing and Analyses

A total of 2 mL of peripheral blood was collected from the proband. Total DNA was extracted using PAXgene Blood DNA Kit (Qiagen, #761133). The library was constructed using Library Preparation Enzymatic Fragmentation Kit (Twist Bioscience). Nucleic acid fragment enrichment and purification kit and GenCap whole exon capture probe kit were used for capture and whole exon sequencing was performed on the BDA‐DNBSEQ‐T7 platform. The effective sequencing data was compared with the reference genome (GRCh37/hg19). In combination with gene variation frequency, clinical database inclusion (ClinVar, HGMD, OMIM), and software prediction results (SIFT, REVEL, MutationTaster, etc.), 4 missense mutations were found in *BRD4* gene. The pathogenesis was rated according to the American College of Medical Genetics and Genomics (ACMG) guidelines.

### Human Islets and *BRD4* Transcript Variants Detection

Fresh human pancreatic islets were purchased from Lonza (#00202998). RNA was extracted by Quick‐RNA Miniprep Kits (Zymo Research Corporation, #R1050). Reverse transcription was performed with 1 µg RNA using the Prime Script RT Reagent Kit (Takara, #RR047A). Primers were chemically synthesized by GenePharma Co., Ltd. (Shanghai, China). Two sets of primers were designed to detect three transcript variants of *BRD4* as follows: TV3‐F1: CCCGACGAGATTGAAATCGACTT; TV3‐R1: GCTCCACATCCACCAGAAACCA; TVS‐F1: TATGTCCTATGAGGAGAAGCGGCAG; TVS‐R1: GCTGGGAAGGAATCTGGAACTGAA; TV3‐F2: CAGCGAAGACTCCGAAACA; TV3‐R2: GCTCCACATCCACCAGAAA; TVS‐F2: CGAAACAGGTCCTGCCTAAT; TVS‐R2: CTGTCCCTTTCACGGAAGAA; TVL‐F: GAAGATCCACTCACCCATCATC; TVL‐R: CGACATCCACAGGCTTCATT. Phusion Green Hot Start II High‐Fidelity PCR Master Mix was purchased from Thermo Fisher (#F566S) and used in PCR according to manufacturer instructions.

### Quantitative Real‐Time PCR (qPCR)

Total RNA was obtained by TRIzol reagent, and RNA concentration and purity were determined by spectrophotometer. Reverse transcription was performed using the Prime Script RT Reagent Kit (Takara, Japan, #RR047A) according to the manufacturer's protocol. RT‒qPCR was conducted with the SYBR Green PCR Kit, and gene expression changes were determined with the comparative CT (2^‐ΔΔCt^) method, and quantification was achieved by normalization using *18s* as control. The primer sequences (5’‐3’) were as follows:

 mouse *Brd4* (CTACGAGAGTTGGAGCGATATG; TCCGACTCAGAGGATGAGAA), human *CHGA* (CCTGTGAACAGCCCTATGAATAA; CCTGTGAACAGCCCTATGAATAA), human *INS* (TCTTCTACACACCCAAGACC; TGGTACAGCATTGTTCCACA), human *PDX1* (CTGCCTTTCCCATGGATGAA; GGAACTCCTTCTCCAGCTCTA), human *PAX6* (GCGGAAGCTGCAAAGAAATAG; GGGCAAACACATCTGGATAATG), human *MAFA* (CGAGAAGTGCCAACTCCAG; CCGCCAGCTTCTCGTATTT), mouse *Atf4* (GGCAAGGAGGATGCCTTT; TGGTTTCCAGGTCATCCATTC), mouse *Atf5* (GCTCGTAGACTATGGGAAACTC; CCCGCTCAGTCATCCAATC).

### Western Blotting

RIPA buffer (Thermo Scientific, #89901) was used to lyse cells to prepare protein extraction. A BCA assay kit (Thermo Scientific, #23227) was used to determine protein concentration. After electrophoresis with SDS‐PAGE, the separated proteins were transferred onto PVDF membrane (0.22 µm, Millipore, USA, #R1PB86935). The PVDF membranes were blocked with 5% skim milk for 1 h at room temperature, and then they were incubated with specific primary antibodies at 4 °C overnight. After incubation with horseradish peroxidase‐conjugated secondary antibodies for 1 h at room temperature, the protein bands were visualized by enhanced chemiluminescence and analyzed by ImageJ software (https://imagej.net/ij/download.html). GAPDH was used as a loading control. The specific primary antibodies were as follows: rabbit anti‐BRD4 antibody (1:1000, Abcam, #ab128874), rabbit anti‐GAPDH antibody (1:3000, Cell signaling, #2118).

### Reporter and Luciferase Assay

The following sequences cloned from the promoter region were used to drive Firefly luciferase expression for luciferase reporter construction: human *ATF4* between −778 and +348 and human *ATF5* between −805 and +514. Luciferase assays were performed with a Dual‐Luciferase® Kit (Promega, #E1960) according to the manufacturer's instructions. Renilla luciferase activities served as internal controls. All reporter plasmids utilized in this study were validated by Sanger sequencing.

### BRD4 Knockdown

Predesigned and validated shRNA lentiviral plasmid for human *BRD4* were purchased from Sigma‐Aldrich (#TRCN0000021427). The target sequence was CCTGGAGATGACATAGTCTTA. A scrambled RNA were used as a negative control, and the sequence of hairpin is: CCTAAGGTTAAGTCGCCCTCGCTCGAGCGAGGGCGACTTAACCTTAGG.

### Immunostaining

The tissues were fixed in 4% paraformaldehyde for at least 24 h, dehydrated and embedded in paraffin. Immunofluorescence staining were performed according to the standard protocols. The primary antibodies were as follows: anti‐Brd4 antibody (1:100; Bethyl laboratories), anti‐Ki67 antibody (1:300; Abcam), anti‐Glut2 antibody (1:100; Abcam), anti‐Mafa antibody (1:100; Abcam), anti‐Proinsulin antibody (1:100; Abcam), anti‐Cre antibody (1:100; Cell signaling Technology), anti‐Somatostatin antibody (1:100; Thermo Fisher), anti‐Glucagon antibody (1:100; Thermo Fisher) and anti‐Insulin (1:300; Abcam). The secondary antibodies for the immunofluorescence staining were purchased from Thermo Fisher. The images were acquired using an Olympus microscopy system.

### Mouse Islets Isolation

2 mL of Hank's buffered saline solution containing 1 mg mL^−1^ type XI collagenase (Sigma) was injected into the pancreas of the mouse models through the bile duct. The pancreas was removed and incubated at 37 °C for 17 min, followed by mechanical dissociation. The islets were hand‐picked under a dissecting microscope. The islets were placed in RPMI 1640 (Invitrogen) culture medium supplemented with 10% (v/v) fetal bovine serum (Invitrogen), 100 U mL^−1^ penicillin and 100 µg mL^−1^ streptomycin (Invitrogen) in a sterile 37 °C incubator with 5% CO_2_ and humidified air for subsequent functional studies. The islets were dissociated using a trypsin EDTA solution (Invitrogen) for sequencing experiments.

### Intraperitoneal Glucose Tolerance Test (IPGTT)

During IPGTT, the mice were fasted for 12 h and injected with 2 g kg^−1^ glucose intraperitoneally. The glucose measurements were taken up to 2 h postinjection using One‐Touch Ultra glucometers (LifeScan). A mouse insulin ELISA kit (Crystal Chem) was used to determine serum insulin levels.

### Single‐Cell Sequencing (scRNA‐Seq)—Primary Analysis of Raw Read Data

Raw reads underwent processing to produce gene expression profiles utilizing CeleScope v1.15.0 (Singleron Biotechnologies), employing default settings. The procedure initially involved extracting and correcting barcodes and UMIs from R1 reads. Subsequently, adapter sequences and poly A tails were removed from R2 reads, which were then aligned to the GRCm38 (mm10) transcriptome using STAR (v2.6.1b). Following unique read mapping, FeatureCounts (v2.0.1) assigned these reads to specific genes. Reads that were successfully allocated, sharing the same cell barcode, UMI, and gene, were aggregated to construct a gene expression matrix for subsequent analytical steps.

### Single‐Cell Sequencing (scRNA‐Seq)—Quality Control, Dimension‐Reduction, and Clustering

Scanpy v1.8.2 was used for quality control, dimensionality reduction, and clustering under Python 3.9. For each sample dataset, the following filtering criteria were imposed on the expression matrix: 1) cells with gene count less than 200 or with top 2% gene count were excluded; 2) cells with top 2% UMI count were excluded; 3) cells with mitochondrial content > 5% were excluded; 4) genes expressed in less than five cells were excluded. Post‐filtering, 89 021 cells remained for subsequent analyses, averaging 2026 genes and 13 683 UMIs per cell. Normalization was conducted by scaling raw counts per cell and applying a logarithmic transformation. Top 2000 variable genes were selected by setting flavor = “seurat.” Principle Component Analysis (PCA) was performed on the scaled variable gene matrix, and top 20 principle components were used for clustering and dimensional reduction. Cells were grouped into 25 clusters via the Louvain algorithm and setting resolution parameter at 1.2. Cell clusters were visualized through Uniform Manifold Approximation and Projection (UMAP).

### Single‐Cell Sequencing (scRNA‐Seq)—Batch Effect Removal

Harmony v1.0^[^ ^32^ ^]^ was utilized to mitigate batch effects between samples using the top 20 principal components from PCA.

### Single‐Cell Sequencing (scRNA‐Seq)—Differentially Expressed Genes (DEGs) Analysis

To detect differentially expressed genes (DEGs), the scanpy.tl.rank_genes_groups() function was employed based on the Wilcoxon rank sum test with default parameters. Genes were considered DEGs if they were expressed in more than 10% of the cells within at least one of the compared cell groups and exhibited an average log(Fold Change) exceeding 1. The statistical significance of these genes was evaluated using an adjusted *p*‐value, calculated through the Benjamini‐Hochberg correction method, with a threshold of 0.05 determining significance.

### Single‐Cell Sequencing (scRNA‐Seq)—Cell Type Annotation

Cell‐type identification was carried out using Cell‐ID, a multivariate method that captures gene signatures for individual cells and applies hypergeometric tests (HGT) for cell identity recognition. Dimensionality reduction was performed on normalized gene expression matrix through multiple correspondence analysis, projecting both cells and genes into a shared low‐dimensional space. Then a gene ranking was calculated for each cell to identify its most prominent gene sets. HGT were performed on these gene sets against brain reference from SynEcoSys database, which contains all cell‐type's featured genes. The cell identity was assigned based on the cell type yielding the minimal HGT *p* value. For cluster annotation, frequency of each cell‐type was calculated in each cluster, and cell‐type with highest frequency was designated as cluster's identity.

### Single‐Cell Sequencing Assay for Transposase‐Accessible Chromatin (scATAC‐seq)—ScATAC Processing and Clustering

Raw sequencing data were converted to fastq format using “cellranger‐atac mkfastq” (10x Genomics, v.2.1.0). scATAC‐seq reads were aligned to the GRCm38 (mm10) reference genome and quantified using “cellranger‐atac count” (10x Genomics, v.2.1.0). Low‐quality cells with less than 1000 sequencing fragments or TSS enrichment less than 4, were excluded from further analysis. Any bin regions overlapping with the ENCODE Blacklist regions were omitted. Next, an iterative latent semantic indexing (LSI) approach were used to reduce the dimensionality of the sparse insertion counts matrix from many thousands to tens or hundreds. To integrate scRNA and scATAC data, canonical correlation analysis (CCA) was applied. Clustering was performed using the addClusters () function in ArchR. Then Uniform Manifold Approximation and Projection (UMAP) was utilized to visualize the data.

### Single‐Cell Sequencing Assay for Transposase‐Accessible Chromatin (scATAC‐seq)—Integration of scRNA and scATAC Data

Utilizing scRNA data as the reference, a classifier was trained to assign cell types to each scATAC cell. FindTransferAnchors() function from the Seurat package facilitated the alignment of data between the two datasets. In the final step of this integration process, for every cell in the scATAC‐seq data, the most similar cell in the scRNA‐seq data was identified, and the corresponding gene expression data from the scRNA‐seq cell was assigned to the scATAC‐seq cell.

### Single‐Cell Sequencing Assay for Transposase‐Accessible Chromatin (scATAC‐Seq)—Peak Calling

MACS2 was used to conduct peak calling based on the aggregated insertion sites from all cells within each cell type. A consensus set of peaks uniform‐length non‐overlapping peaks was generated by selecting the peak with highest score from each set of overlapping peaks. In brief, the peaks were ranked according to their significance, retained the most significant peak, and excluded any overlapping peaks from further consideration. This process was iteratively applied to the remaining peaks until no more peaks were left.

### Single‐Cell Sequencing Assay for Transposase‐Accessible Chromatin (scATAC‐Seq)—ScATAC Gene Score/Transcription Factor Activity Analysis

ArchR was used to estimate gene expression (also named gene scores) for genes and TF motif activity from scATAC data. Gene scores were calculated using the addGeneScoreMatrix() function with gene score models implemented in ArchR. For identifying motif occurrences within the peak set, the addMotifAnnotations() function was used in conjunction with the JASPAR2020 motif dataset. Then, addDeviationsMatrix() function was applied to compute enrichment of TF activity on a per‐cell basis across all motif annotations based on chromVAR.

### Single‐Cell Sequencing Assay for Transposase‐Accessible Chromatin (scATAC‐Seq)—Public ChIP‐Seq and Human Islet Single‐Nuclear Sequencing (snRNA‐Seq) Data Analysis

Processed BED and WIG files were download from ChIP‐atlas (http://ChIP‐atlas.org/).^[^ ^33^ ^]^ WIG files were opened in integrative genomics viewer (IGV) to obtain the “PEAK” graph. CLC Genomics Workbench 12 was used to extract the target gene information about “PEAK score,” annotation and nearby gene information. SnRNA‐seq matrix files for human islets from nondiabetic, prediabetes and type 2 diabetes donors were downloaded from NCBI GEO (GSE200044).^[^ ^22^ ^]^

### Statistical Analysis

All of the data were presented as the mean ± SEM. Differences between the groups were evaluated using unpaired Student's *t*‐test or one‐way ANOVA followed by Tukey's test via GraphPad Prism 8 software. *p* < 0.05 was considered to be statistically significant.

## Conflict of Interest

The authors declare no conflict of interest.

## Supporting information



Supporting Information

## Data Availability

The data that support the findings of this study are available in the supplementary material of this article.
