# Supplementary material for: BRD4 Signaling Maintains the Differentiated State of β Cells
Source: Adv Sci (Weinh). 2025 Jun 20;12(33):e05659. doi: 10.1002/advs.202505659 (PMC12412598; doi:10.1002/advs.202505659)
Supplement: Supplementary file 1 — Supporting Information [file ADVS-12-e05659-s001.pdf]

ADVANCED SCIENCE
Open Access

Supporting Information

for *Adv. Sci.*, DOI 10.1002/advs.202505659

BRD4 Signaling Maintains the Differentiated State of β Cells

Fuqiang Liu, Guang Liu, Jia Song, Yujing Sun, Mengmeng Yang, Hualin Liu, Hongkai Zhao, Jiamu Chen, Qincheng Qiao, Siyue Li, Chenglong Yu, Jingru Qu, Ying Zou, Tixiao Wang, Jidong Liu, Lei Zhao, Huihui Tian, Tao Huang, Manna Zhang, Li Chen, Ruxing Zhao, Yuanyuan Du* and Xinguo Hou**

Supplementary Table

Table S1. Clinical characteristics of young diabetes patients for WES experiments.

	Mean(SD) Median (Min-Max)
Age(years)	23.49(13.01) 19.00(5.00-68.00)
BG 0h	10.22(5.63) 8.50(4.20-48.83)
BG 2h	13.89(6.62) 12.50(4.08-31.44)
CP 0h	2.80(15.22) 1.14(0.03-173.00)
CP 2h	12.69(86.78) 2.31(0.26-817.00)
INS 0h	25.73(46.25) 10.67(1.55-324.79)
INS 2h	94.72(291.81) 29.90(1.53-2171.00)
**Gender**	
Male	115(51.80%)
Female	107(48.20%)

BG, blood glucose. CP, C peptide. INS, blood insulin. 0h and 2h, different time points in OGTT experiments.

Supplementary Figures

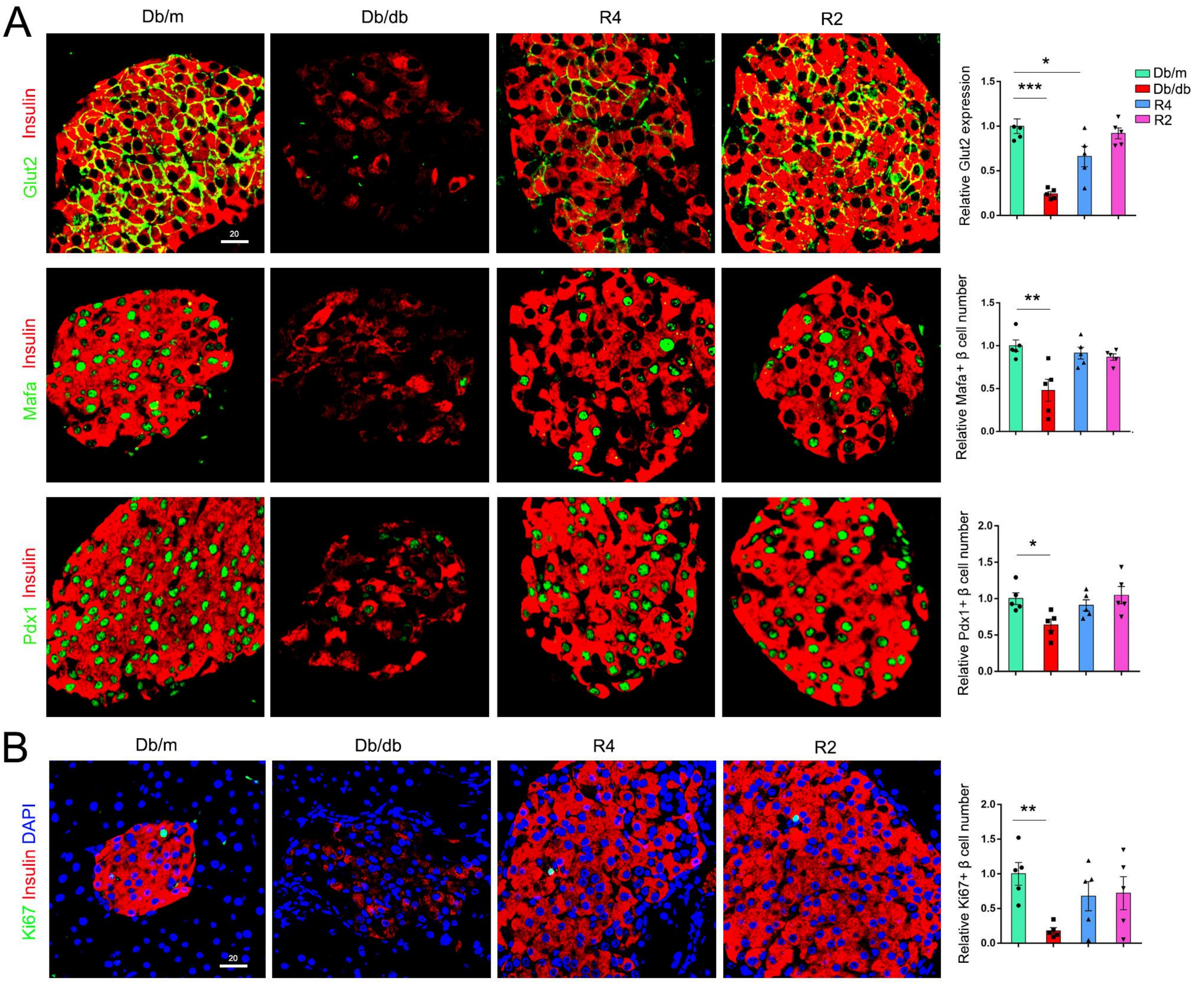

Figure S1. *Brd4* expression is related to the differentiation of β cells in a caloric restriction mouse model. Representative images of immunofluorescence staining demonstrating Glut2 (green), Mafa (green), Pdx1 (green), insulin (red) and DAPI (blue) in the islets from db/db, db/m, R4 and R2 mice (n=5, A). Representative images of immunofluorescence staining demonstrating Ki67 (green), insulin (red) and DAPI (blue) in the islets from the different groups (n=5, B). Scale bar=20μm.

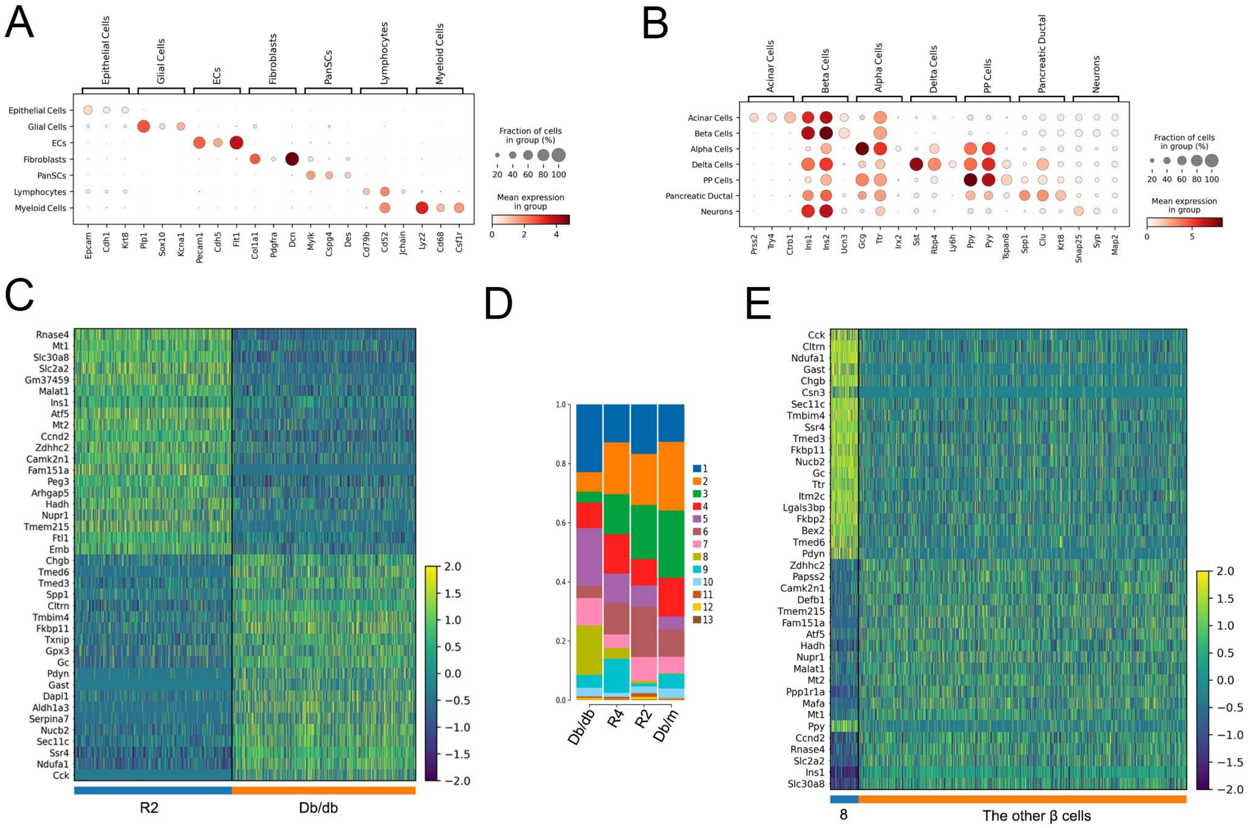

Figure S2. Gene expression differences in db/db, db/m, R4 and R2 β cells. Cell type marker genes that were used to identify the clusters generated via UMAP plotting (A-B). Heatmap of the top 20 significant DEGs that were up- or downregulated in R2 and db/db β cells. The data are colored according to the expression level, and the legend is labeled on a log scale (C). The mean proportion of each cell subtype is presented for epithelial cells in the db/db, db/m, R4 and R2 islet samples (D). Heatmap of the top 20 significant DEGs that were up- or downregulated in subtype 8 and the other β cells (E). ECs, Endothelial cells.

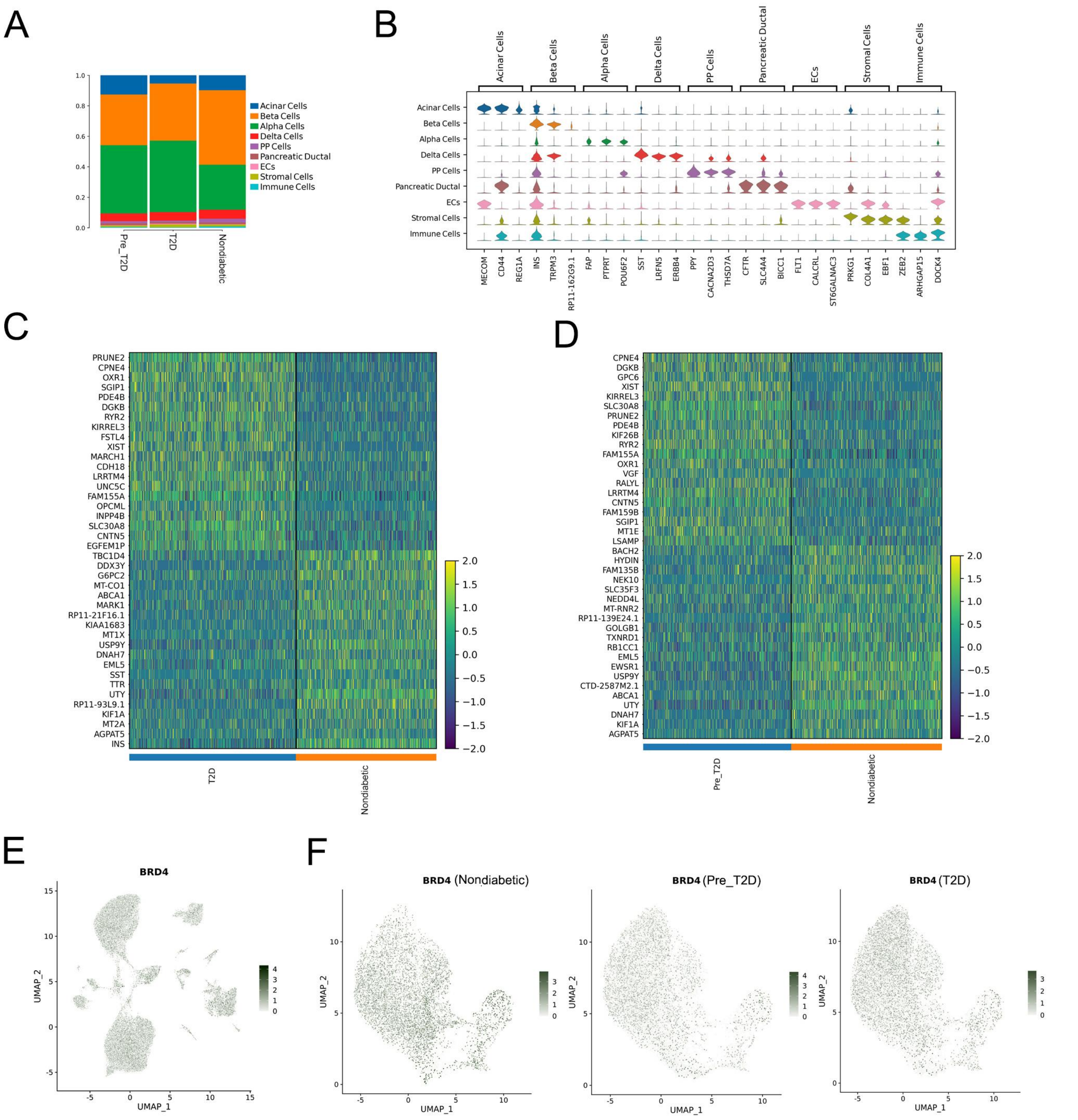

Figure S3. Gene differences in nondiabetic, prediabetic (pre_T2D), and type 2 diabetes (T2D) β cells. The mean proportion of each cell subtype is presented for islet cells in nondiabetic, pre_T2D and T2D islet samples (A). Violin plot of cell type marker genes that identified the clusters generated by UMAP plotting (B). Heatmap of the top 20 significant DEGs up- or downregulated in T2D (C) and pre_T2D (D) β cells compared with nondiabetic β cells. The data are colored according to expression level, and the legend is labeled on a log scale. Feature plot of BRD4 expression in islet cells (E). β cells were reclustered and feature plot shows the BRD4

expression in three groups (F). The data are colored according to expression level, and the legend is labeled on a log scale.

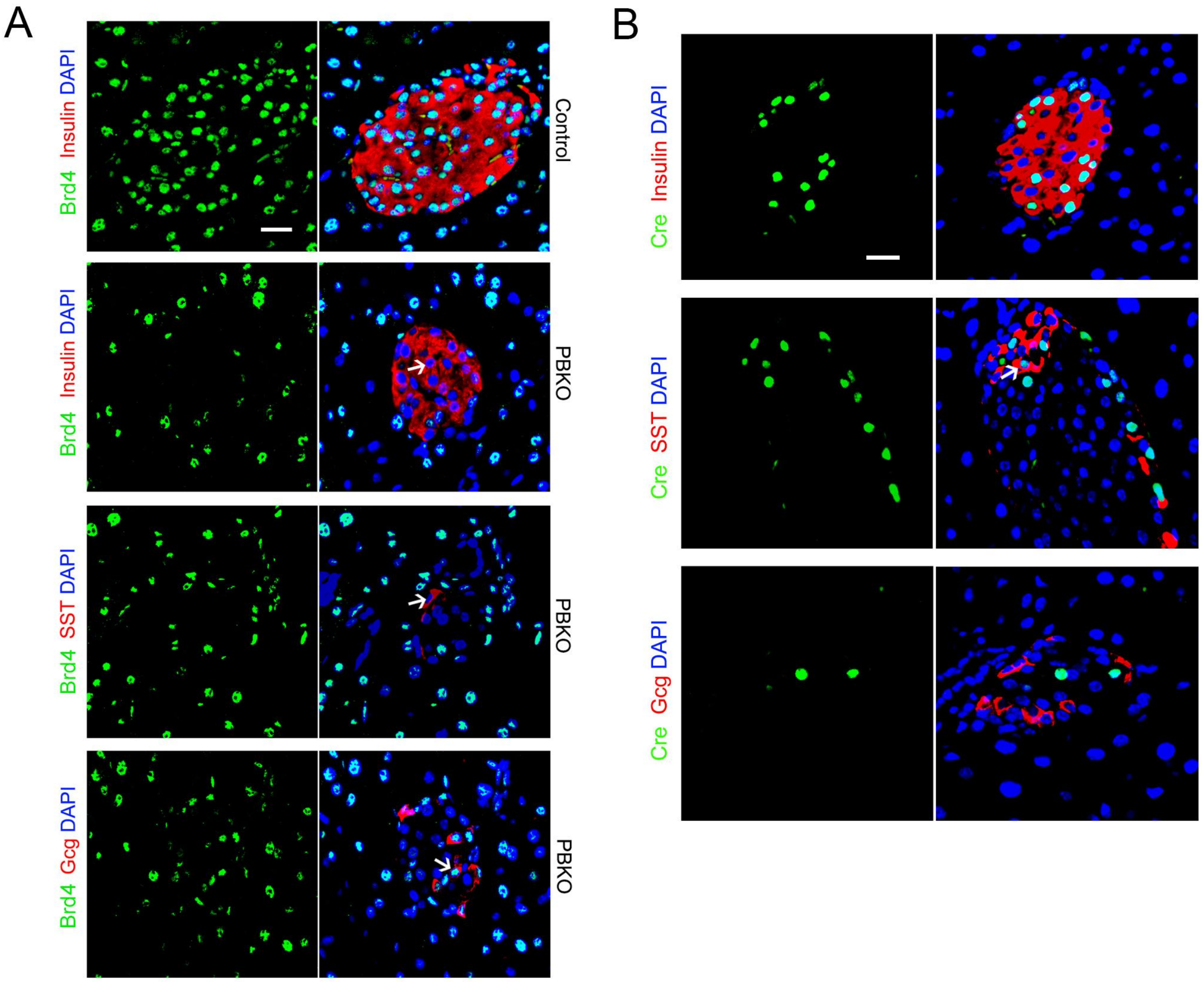

Figure S4. Acute deletion of *Brd4* in β cells. Representative IF images depicting Brd4 (green) and Insulin (red), Somatostatin (SST, green), and Glucagon (Gcg, red) in pancreatic islets from control and RBKO mice. Arrows indicate the expression of Brd4 (A). Representative IF images depicting Cre (green) and Insulin, Somatostatin (SST, green), and Glucagon (Gcg, red) in pancreatic islets from RBKO mice. Arrows indicate the expression of Cre (B).

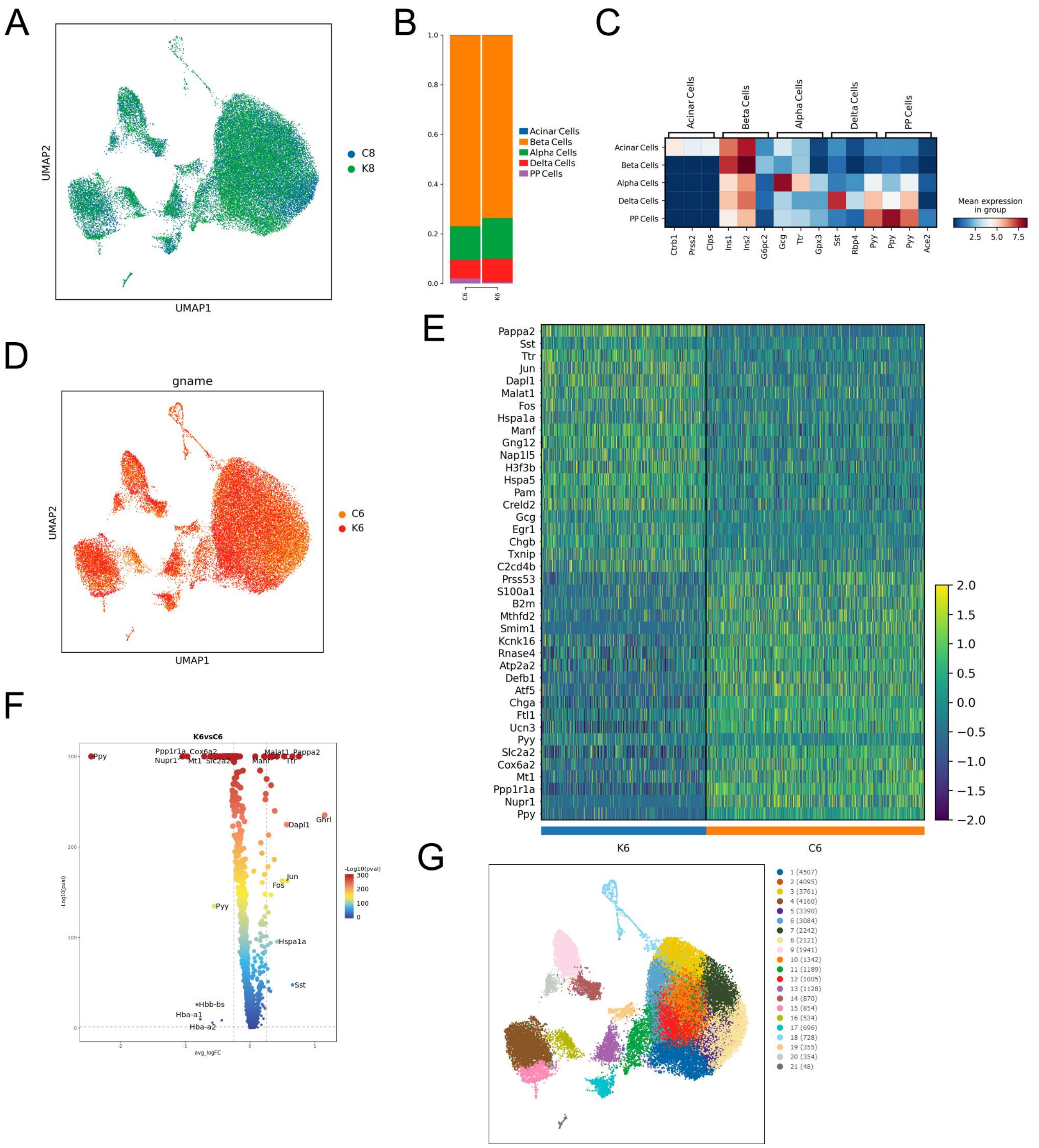

Figure S5. Changes in PBKO β cells indicated by scRNA-seq. UMAP visualization of the single-cell transcriptomic dataset of islet C8 and K8 cells. Different colors represent different samples (A). The mean proportion of each cell subtype is presented for C6 and K6 (B). Cell type markers for C6 and K6 islet cell clustering (C). UMAP visualization of the single-cell transcriptomic dataset of islet C6 and K6 cells. Different colors represent different samples (D). Heatmap of the top 20 significant DEGs up- or downregulated in the C6 and K6 β cells. The data

are colored according to expression level, and the legend is labeled on a log scale (E). Volcano plot depicting significant DEGs between K6 and C6 β cells. The data are colored according to expression level, and the legend is labeled on a log scale (F). Twenty-one cell subtypes were identified in the UMAP plot for C6 and K6 islet cells (G). C8, control mice at eight weeks of age. K8, knockout mice at eight weeks of age. C6, control mice at six weeks of age. K6, knockout mice at six weeks of age.

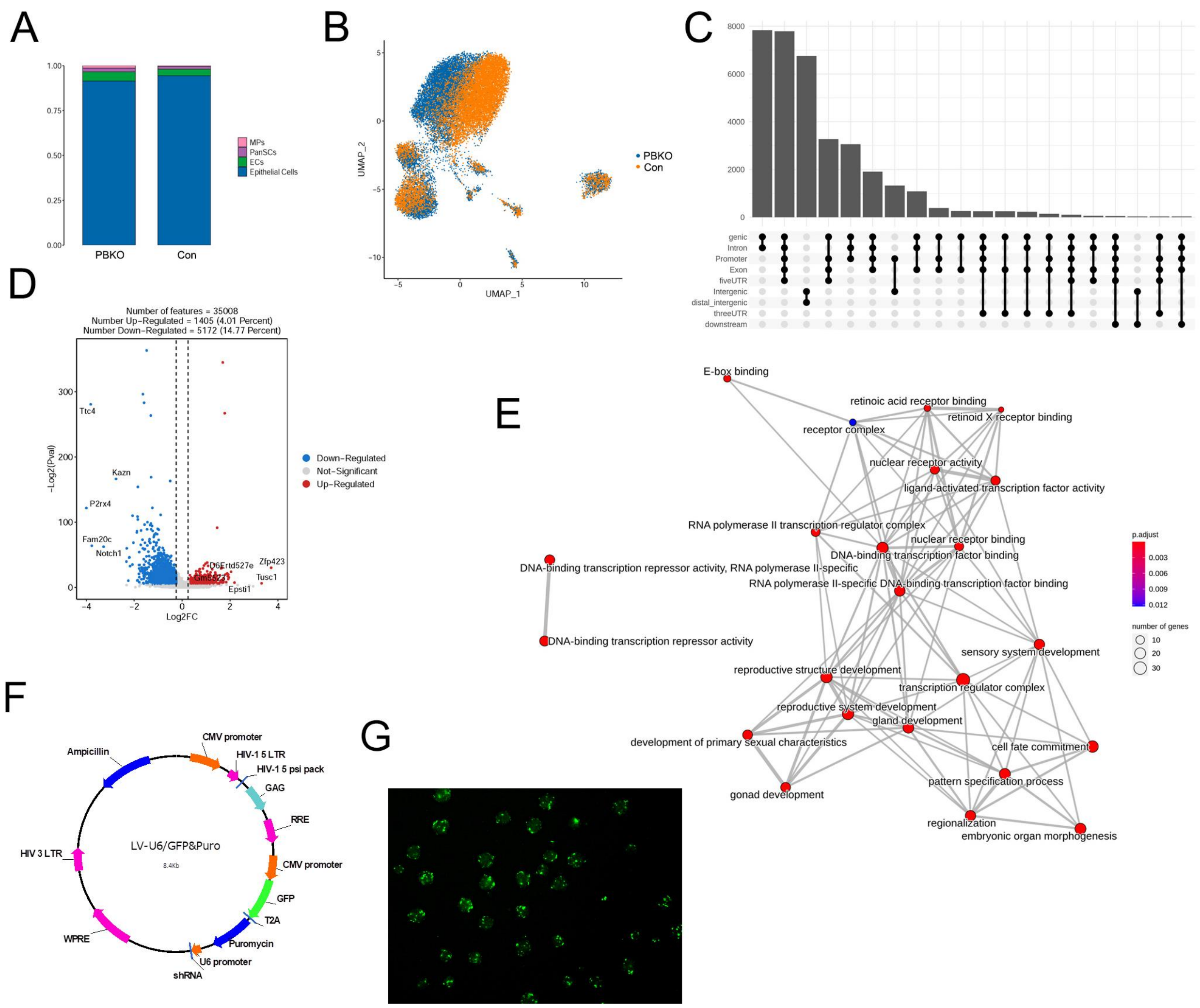

Figure S6. Changes in PBKO β cells indicated by scATAC-seq. Proportion of distinct cells identified by different chromatin accessibility patterns within transcription factor start sites (TFSSs) (A). Clustering of the cells in different groups based on chromatin accessibility patterns within the TFSS (B). Upset plot showing the overlap of a given peak at the gene level in PBKO

and control β cells from the ATAC datasets (C). Volcano plot depicting DEGs based on changes in chromatin accessibility after Brd4 knockout in β cells (D). KEGG enrichment analysis of differentially expressed transcription factors identified by transcription factor activity analysis (E). The plasmid map of the BRD4 shRNA; GFP expression was driven by a separate CMV promoter (F). The representative image showing the infection efficiency of BRD4-shRNA lentivirus in human islet organoids. The image was taken on a microscope with a magnification of 4x to show a larger field of view. Green is GFP signal (G). Con, control.

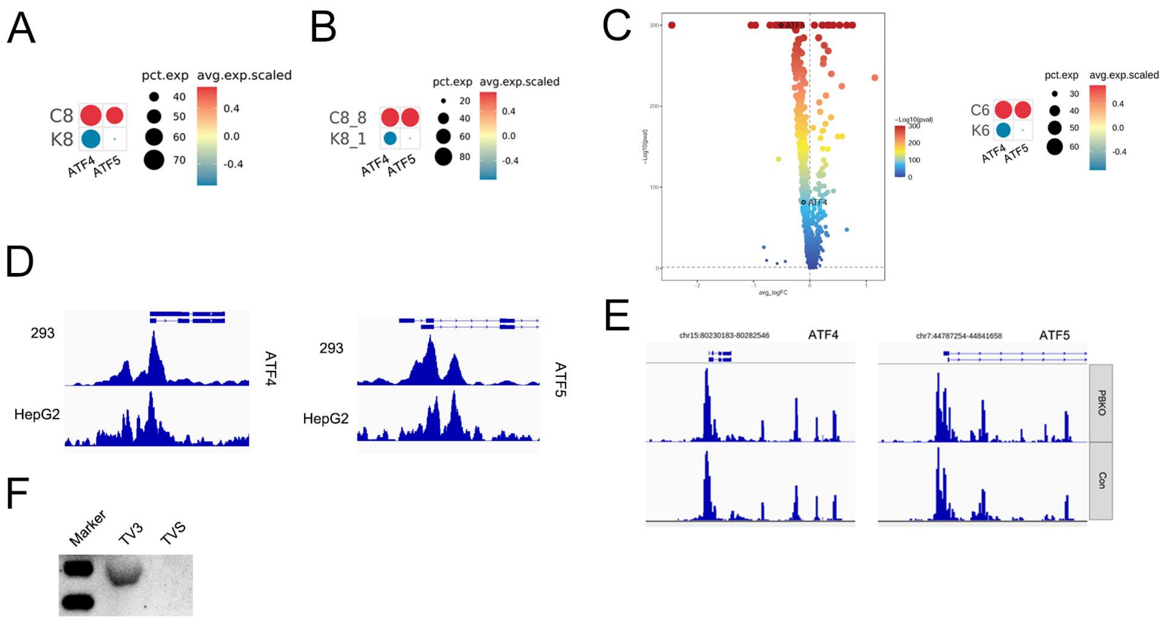

Figure S7. ATF4 and ATF5 can be directly regulated by BRD4. Bubble diagram illustrating the *Atf4* and *Atf5* gene expression levels in C8 and K8 β cells. The color depth and size of each bubble were positively correlated with *Atf4* and *Atf5* expression, respectively (A). Bubble diagram illustrating the *Atf4* and *Atf5* gene expression levels in subtype 1 K8 (K8_1) and subtype 8 C8 (C8_8) β cells. The color depth and size of each bubble were positively correlated with *Atf4* and *Atf5* expression, respectively (B). Volcano plot and bubble diagram depicting the reduction in *Atf4/Atf5* expression in K6 cells compared with that in C6 β cells. The data are colored according to expression level, and the legend is labeled on a log scale (C). IGV visualization of BRD4 binding peaks at the promoter regions of *ATF4* and *ATF5* in 293 and HepG2 cells based on ChIP-seq data (D). IGV visualization of chromatin accessibility peaks in the *Atf4* and *Atf5*

promoter regions in PBKO and control β cells (E). PCR detection of TV3 and TVS in human islets by high-fidelity DNA polymerases with another set of primers (F). TVL, transcript variant for long isoform of BRD4. TVS, transcript variant for short isoform of BRD4. TV3, BRD4 transcript variant 3.
